# Synthesis and Anticancer Activity of Mitotic-Specific 3,4-Dihydropyridine-2(1*H*)-thiones

**DOI:** 10.3390/ijms22052462

**Published:** 2021-02-28

**Authors:** Magdalena Perużyńska, Aleksandra Borzyszkowska-Ledwig, Jacek G. Sośnicki, Łukasz Struk, Tomasz J. Idzik, Gabriela Maciejewska, Łukasz Skalski, Katarzyna Piotrowska, Paweł Łukasik, Marek Droździk, Mateusz Kurzawski

**Affiliations:** 1 Department of Experimental and Clinical Pharmacology, Pomeranian Medical University in Szczecin, Powstanców Wielkopolskich 72, 70-111 Szczecin, Poland; lukasz.skalski@pum.edu.pl (Ł.S.); marek.drozdzik@pum.edu.pl (M.D.); mateusz.kurzawski@pum.edu.pl (M.K.); 2 Department of Organic and Physical Chemistry, Faculty of Chemical Technology and Engineering, West Pomeranian University of Technology, Szczecin, Al. Piastów 42, 71-065 Szczecin, Poland; aleksandra.borzyszkowska@zut.edu.pl (A.B.-L.); Jacek.Sosnicki@zut.edu.pl (J.G.S.); Lukasz.Struk@zut.edu.pl (Ł.S.); Tomasz.Idzik@zut.edu.pl (T.J.I.); 3 Faculty of Chemistry, Wrocław University of Technology, Wybrzeże Wyspiańskiego 27, 50-370 Wrocław, Poland; gabriela.maciejewska@pwr.edu.pl; 4 Department of Physiology, Pomeranian Medical University in Szczecin, Powstanców Wielkopolskich 72, 70-111 Szczecin, Poland; katarzyna.piotrowska@pum.edu.pl; 5 Department of Medical Chemistry, Pomeranian Medical University in Szczecin, Powstanców Wielkopolskich 72, 70-111 Szczecin, Poland; pawel_lukasik@yahoo.co.uk

**Keywords:** dihydropyridine, mitotic spindle, tubulin inhibitors, colchicine-binding site, anticancer agents

## Abstract

Most anticancer drugs target mitosis as the most crucial and fragile period of rapidly dividing cancer cells. However the limitations of classical chemotherapeutics drive the search for new more effective and selective compounds. For this purpose structural modifications of the previously characterized pyridine analogue (**S1**) were incorporated aiming to obtain an antimitotic inhibitor of satisfactory and specific anticancer activity. Structure-activity relationship analysis of the compounds against a panel of cancer cell lines allowed to select a compound with a thiophene ring at C5 of a 3,4-dihydropyridine-2(1*H*)-thione (**S22**) with promising antiproliferative activity (IC_50_ equal 1.71 ± 0.58 µM) and selectivity (SI = 21.09) against melanoma A375 cells. Moreover, all three of the most active compounds from the antiproliferative study, namely **S1**, **S19** and **S22** showed better selectivity against A375 cells than reference drug, suggesting their possible lower toxicity and wider therapeutic index. As further study revealed, selected compounds inhibited tubulin polymerization via colchicine binding site in dose dependent manner, leading to aberrant mitotic spindle formation, cell cycle arrest and apoptosis. Summarizing, the current study showed that among obtained mitotic-specific inhibitors analogue with thiophene ring showed the highest antiproliferative activity and selectivity against cancer cells.

## 1. Introduction

The key function of microtubules and mitotic spindles in cell division make them an attractive target in cancer therapy. Therefore, microtubule-targeting agents (MTAs), also named “spindle poisons” remain the most important of classical chemotherapeutics [1]. These drugs are divided into two main groups: microtubule stabilizing agents (including taxanes, epothilones, laulimalide) and microtubule destabilizing agents (colchicine, vinca alkaloids, combretastatins) with five different tubulin binding sites [2]. Microtubule polymerization and likewise depolymerization are necessary for correct cell division. Therefore, despite different actions, both types of MTAs disrupt microtubule dynamics, leading to aberrant mitotic spindle formation, cell cycle arrest at G2/M phase, activation of the mitotic spindle assembly checkpoint (SAC) and apoptosis [3]. However, classical MTAs are characterized by a narrow therapeutic index and reported resistance of some cancer cells, which encourages a search for new more selective and effective compounds. Most efforts are focused on the new colchicine-binding site inhibitors (CBSIs), because of their ability to overcome P-glycoprotein (P-gp)/class III β-tubulin mediated drug resistance and antiangiogenic/antivascular actions [4]. Colchicine, a prototype drug among CBSIs, that binds to tubulin with high affinity, is not used as an anticancer agent, mainly because of its very narrow therapeutic index [5,6]. Moreover, in spite of the fact hundreds of promising CBSIs have been tested in vitro and in vivo, there are no approved colchicine-binding site drugs for the treatment of cancer. Simultaneously with the search of new potential CBSIs, many efforts are made to obtain small molecules that target kinesins and kinases involved in mitotic spindle organization [7,8]. 

Our previously published data showed that 4-benzyl-5-phenyl 3,4-dihydropyridine-2(1*H*)-thione (**S1**) demonstrated promising antiproliferative activity, with observed cell cycle arrest at G2/M phase, aberrant mitotic spindle formation and inhibition of tubulin polymerization [9]. Pyridine (with piperidine) are two of the most common heterocyclic fragments found in Food and Drug Administration (FDA) approved drugs [10]. Interest in the anticancer activity of dihydropyridines (DHP) and structurally related dihydropyrimidines (DHPM) has been increasing since monastrol was first described [11]. Monastrol is the first known specific inhibitor of kinesin-5 (Eg-5), a motor protein involved in centrosome separation and bipolar spindle formation, required for correct chromosomes segregation [12]. However, monastrol anticancer activity is not satisfactory, encouraging research for more active analogues [13]. Kumar et al., using comparative molecular field analysis (CoMFA), showed that 1,4-dihydropyridine and 1,4-dihydropyrimidine motifs being bioisosteric pharmacophores are common for both compounds and required for their cytotoxicity [14]. 

**Figure 1:**
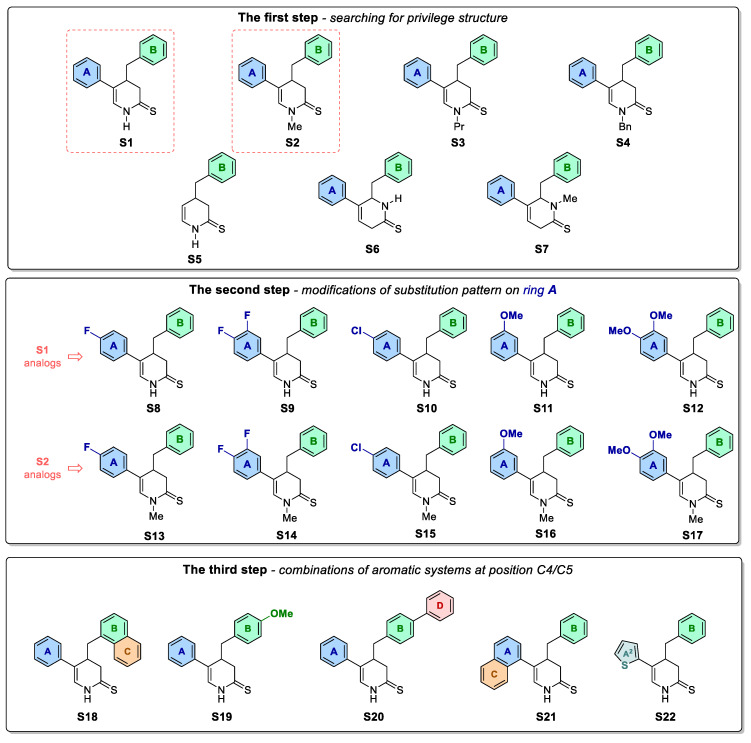
Compounds **S1**-**S22** obtained and tested within a three-step optimisation process.

As we found our previously published results quite promising, we conducted further derivatization of the pyridine analogue **S1** (Figure 1) aimed to obtain antimitotic inhibitor of satisfactory and specific anticancer activity. For this purpose, series of structural modifications followed by the antiproliferative study were conducted according to three stages: (1) selection of the most active compounds from N-H and N-substituted derivatives of the compound **S1**; (2) derivatization of the most potent compound from the first stage by adding appropriate substituents to A ring; (3) further modifications of aryl substituents at C4/C5 of 3,4-dihydropyridine-2(1*H*)-thione. Afterwards, mitotic-specific activity of selected compounds were evaluated against A375 melanoma cell line.

## 2. Results and Discussion

### 2.1. Synthesis of Dihydropyridine-2-thiones **S1**-**S22**

**Scheme 1:**
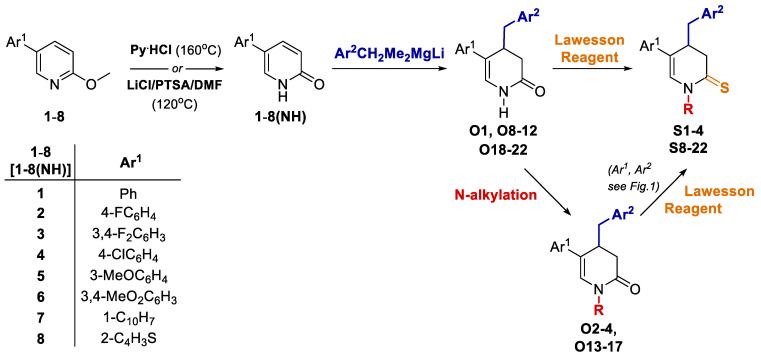
Sequence of the synthesis of targeted compounds **S1**-**S4** and **S8**-**S22**.

Compounds **S1**-**S4** and **S8**-**S22** (Figure 1) were obtained according to the sequence of reactions depicted in Scheme 1. It comprises demethylation of 5-aryl-2-methoxypyridines **1**-**8** by heating with the mixture of PTSA and LiCl or with pyridinium hydrochloride leading to corresponding 5-aryl NH 2-pyridones **1**(**NH**)-**8**(**NH**). Starting compounds **1**-**8** were obtained mainly through the cross coupling reactions between 5-bromo-2-methoxypyridine and corresponding arylboronic acids in the presence of PdCl_2_ as catalyst. The key reaction consisted of the addition of ArCH_2_Me_2_MgLi complex to NLi 2-pyridones of **1**(**NH**)-**8**(**NH**), yielding **O1**, **O6** (not seen, see Appendix A) **O8**-**O12** and **O18**-**O22**. Subsequently, part of obtained 4-benzylated 3,4-dihydropyridin-2-ones were next transformed to N-substituted derivatives **O2**-**O4** and **O13**-**O17**. Finally, both NH and N-substituted 3,4-dihydropyridin-2-ones were transformed to their sulphur analogues **S1**-**4** and **S8**-**22** by thionation using Lawesson’s reagent. Compounds **S5** was obtained by addition of benzylmagnesiate to 2-thiopyridone as described earlier [15], while compound **S7** was achieved by thionation of oxygen analogue **O7**, obtained by addition of benzylmagnesiate to N-Me 5-phenylpyridin-2-one. Product **S6** was gained by thionation of oxygen analogue **O6**, obtained as by-product during benzylation conducted with bigger amount of MeLi in comparison to a standard procedure. The structures of all compounds were elucidated with the aid of 1D NMR (^1^H, ^13^C, ^13^C-DEPT-135) and basic 2D NMR spectroscopy, low (GC-MS) and high (HRMS) resolution mass spectrometry and elemental analyses. The dihydropyridinones (**O**-serie) and dihydropyridinethiones (**S**-serie) were obtained in the form of racemates. Synthesis and spectroscopic data of **S1**-**S4** and **S6**-**S22** is described in the Materials and methods (4.1. Experimental part), while synthesis and spectroscopic data of their precursors is presented in the Appendix A (Synthesis of precursors of compounds **S1**-**S22**).

### 2.2. The Antiproliferative Activity

The compound **S1** with previously described promising anticancer activity [9] has been chosen as a starting point for the current study. It should be emphasized that biological activity of **S1** was evaluated at single high concentration (100 µM) in the previous study. The Cell Proliferation Reagent (WST-1 assay) was used to evaluate the antiproliferative activity of **S1** and its analogues against selected cancer cell lines including breast (MCF7), melanoma (A375), colon (HT-29), ovarian (SK-OV-3) and prostate (PC-3). A combretastatin (CA-4) one of the most extensively researched CBSIs [16], was used as reference control as it was predicted that **S1** may bind to colchicine-binding side (which is presented and discussed below). The results are summarized as IC_50_ values in Table 1. The data revealed that from the following N-H and N-substituted derivatives compound **S1** with N-H moiety and **S2** with N-Methyl group were the most promising (IC_50_ values equal 4.33 ± 1.00 µM and 12.55 ± 2.16 µM against A375, respectively). Moreover, structure-activity relationship study of the first step showed that benzene ring is essential for the cytostatic activity of the studied structure since the analogue without 5-phenyl group (**S5**) lost anticancer activity (IC_50_ > 100 µM). The importance of that group was previously confirmed in case of dihydropyrimidinethiones derivatives [17]. In spite of increase lipophilic properties, compounds **S6** and **S7** (isomers of **S1** and **S2**, respectively) were markedly less active, probably as a result of different position of nitrogen atom in relation to phenyl group. 

During the second task of the study compounds **S1** and **S2** were further derivatized by adding appropriate groups to A-ring. Selected groups were introduced on the basis of their π parameter, determining influence of different substituents on Log *p* value: two derivatives (**S8**/**S13** and **S9**/**S14**) containing respectively one or two fluorine atoms (each of F atoms increases Log P by 0.13), one derivative (**S10**/**S15**) containing chlorine, increasing Log *p* value by 0.51, two derivatives (**S11**/**S16** and **S12**/**S17**) containing one or two methoxy groups, respectively (each of the groups decreases Log *p* value by 0.26 compared to the original compound). In the present study we did not observe direct correlation between Log *p* values and antiproliferative activity, as it was noted in our previously published data, where the most lipophilic pyrimidine analogues were also the most potent growth inhibitors [17]. The introduction of substituents at the para-position of the phenyl ring (compounds **S8**-**S17**) resulted in a reduction of antiproliferative activity against all selected cancer cell lines, as compared with the activity of the unsubstituted A-ring analogue (**S1** and **S2**). 

When the effect of electron-releasing (ERG’s) or electron-withdrawing groups (EWG’s) at the phenyl ring was compared, compounds **S11** and **S16** with electron-donating methoxy groups were generally the most potent among –N-H and –N-Me analogues, respectively. Our data are compliant with Romagnoli et al. study, where the presence of electron-donating group (like methyl, methoxy) rather than electron-withdrawing groups on the phenyl moiety at the position of the 2-alkoxycarbonyl-3-(3′,4′,5′-trimethoxyanilino)thiophene system contributed to increase biological activity [19]. Surprisingly, in the current study two methoxy group (**S12** and **S17**) contributed to decrease anticancer potential. In general, during the second stage of the study, -N-H analogues (**S8**-**S12**) were more potent than -N-Me counterparts (**S13**-**S17**). Therefore, the antiproliferative activity of -N-H derivatives with aromatic rings at C4/C5 position were evaluated at the third stage. As shown in Table 1, additional modifications of aryl rings at C4 and C5 contributed to increased anticancer activity (IC_50_ values of compounds **S18**-**S22** ≤ 10 µM), however, an analogue with thiophene ring at C5 pyridine-2(1*H*)thione (**S22**) was the most effective one, with IC_50_ value of 1.71 ± 0.58 µM and 1.67 ± 1.47 µM against A375 and SK-OV-3 cell line, respectively. These findings correspond with several previous studies where thiophene, benzothiophene and fused thienopyridine or thienopyrimidine rings are presented in a variety of anticancer compounds [19,20,21], including CBSIs [22,23,24].

### 2.3. Selectivity-Index

The starting compound (**S1**) and its two derivatives defined as the most active compounds in the antiproliferative study (**S19** and **S22**) were selected for further biological study, using A375 cells, that presented the highest sensitivity to the tested compounds. At first, the antiproliferative assay (WST-1) was used to estimate the selectivity-index (SI) defined as ratio of IC_50_ for normal cells to IC_50_ for cancer cells. Cancer cell line A375 and a non-tumor human melanocyte cell line (HEM) were used for that purpose. As shown in Table 2, all the newly evaluated compounds showed better selectivity against cancer cells when compared with reference: CA-4. Similar to our study, Amaral et al. presented CA-4 as non-selective cytotoxic agent. Namely, the authors revealed that CA-4 showed higher antiproliferative activity against human lymphocytes than tumor cell lines, except for HL-60 and OVCAR-8 [25]. Whereas, Blay et al. reported that in spite of the combination of ombrabulin (CA-4 analogue) and cisplatin significantly improved progression-free survival in patients with advanced soft-tissue sarcomas, serious adverse effect excluded this strategy from anticancer therapy [26]. According to the “selectivity criteria” (SI > 10 [27]) only the compound **S22** could be considered as selective. Similarly, Hura et al. revealed that among twenty-one tested CA-4 derivatives, C-13 analogue with thiophene as ring B was the most potent and selective against cancer cells [24].

### 2.4. Apoptosis Detection 

**Figure 2:**
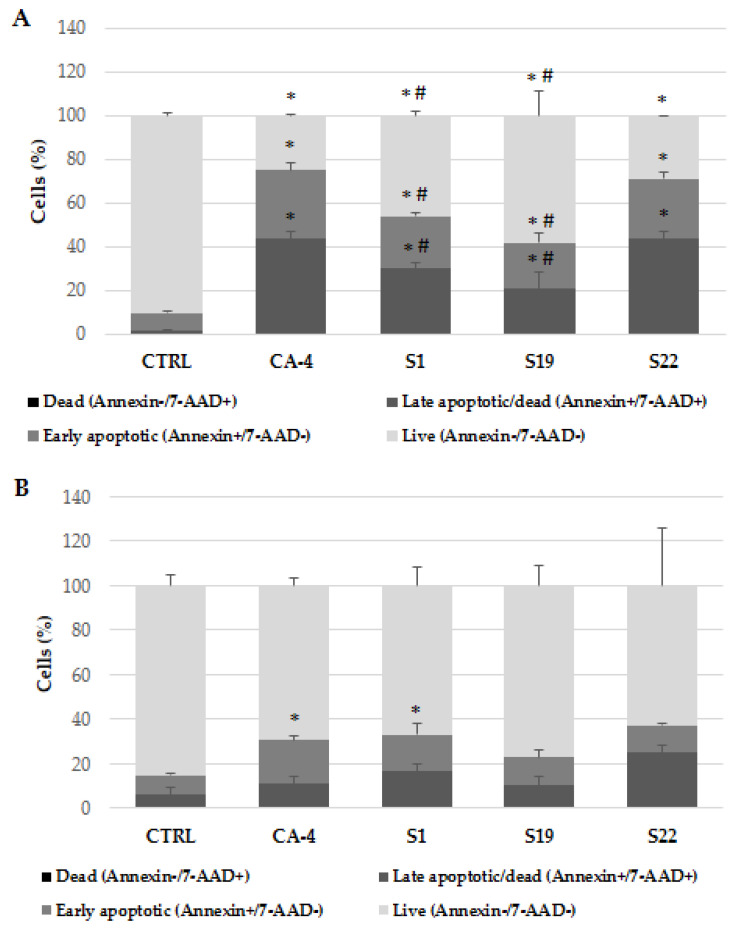
The effect of indicated compounds (at 10 µM) on the induction of apoptosis in A375 (**A**) and HEM (**B**) cells after 48 h treatment, subsequently stained with Annexin V-PE and 7-AAD and analyzed by flow cytometry. Data are expressed as the mean and SD from at least three independent experiments; * *p* < 0.05 vs. ctrl; # *p* < 0.05 vs. CA-4.

Since apoptosis is identified as one of the most common mechanism of drug induced cell death, an apoptotic marker was evaluated using flow cytometry in the next step of the study. The assay used was based on the detection of phosphatidylserine (PS) on the surface of apoptotic cells. A dead cell marker (7-AAD) is also used as an indicator of the cell membrane structural integrity. Simultaneous staining with two dyes allows to distinguish (after debris excluding) viable (Annexin V-PE− and 7-AAD−), early apoptotic (Annexin V-PE+ and 7-AAD−) and late apoptotic/already dead cells (Annexin V-PE+ and 7-AAD+). The A375 and HEM cells were treated with the compounds: **S1**, **S19** and **S22** at 10 µM for 48 h. CA-4 was used as reference. As shown in Figure 2A, the population of living (non-apoptotic) cancer cells significantly decrease after treatment. Moreover, compound **S22** showed the most comparable pattern to CA-4 with the percentage of early and late apoptotic cells equal to 27.42 ± 3.20% and 43.58 ± 3.30% for **S22** and 31.57 ± 3.16% and 43.42 ± 3.43% for CA-4, in A375 and HEM cells, respectively. As for **S1**, **S19** and **S22**, more apoptotic cells could have been expected after treatment with 10 µM concentration (based on IC_50_ values from WST-1 assay), obtained data from apoptosis test suggest that those compounds possess stronger antiproliferative than direct cytotoxic potential. Simultaneously, analysis was performed in non-cancerous cells what allowed to confirm selectivity observed during antiproliferative study (Figure 2B). Namely, only CA-4 and **S1** significantly increased the percentage of early apoptotic cells to 19.74 ± 2.19% and 16.43 ± 4.88%, respectively when compared to untreated control (8.8 ± 1.1%). Surprisingly, during apoptosis detection in both cell lines, the differences between reference CA-4 and our compounds seemed less distinct than during IC_50_ evaluation. Representative individual experiments (scatter diagrams with cell populations divided into quadrant dot plots specific for Annexin V/7-AAD staining) are shown in Figures S1 and S2. Additionally, optical microscopy imaging of A375 and HEM cells was performed just before apoptosis analysis of harvested cells. As shown in Figure S3, A375 treated cells had lower density in comparison with non-treated cells, while increased the number of dead cells could be observed only in CA-4 and **S22** exposed cells, which was consistent with flow cytometry results. In the HEM cells, the most striking difference between control and treated cells was in cell morphology, especially in case of CA-4 and **S22** exposed cells (Figure S4).

### 2.5. Cell Cycle Analysis 

**Figure 3:**
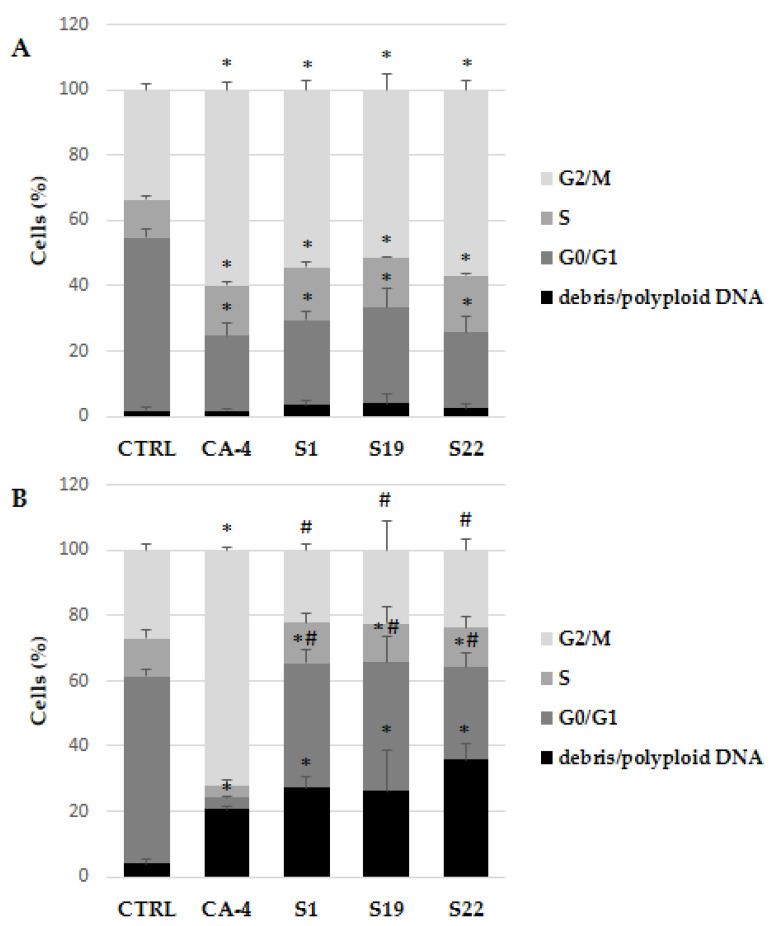
Flow cytometry analysis of cell cycle in A375 cells using PI staining after exposure to indicated compounds (at 10 µM) for 6 h (**A**) and 24 h (**B**). Data are expressed as the mean and SD from three independent experiments; * *p* < 0.05 vs. ctrl; # *p* < 0.05 vs. CA-4.

Since growth inhibition may be a result of arrest cell cycle at a particular point, specific antiproliferative activity of the selected compounds was further evaluated by flow cytometry, for the impact on the cell cycle progression in A375 cells. The assay utilizes PI-based staining of DNA content to discriminate and measure the percentage of cells in each cell cycle phase (i.e., G0/G1, S, and G2/M) [28]. In present study, the cells were treated with tested compounds at 10 µM concentration for 6 and 24 h, aimed to detect cell cycle arrest leading to apoptosis at 48 h of treatment. As expected, **S1**, **S19** and **S22** compounds caused accumulation of A375 cells in a tetraploid (4N) state, simultaneously decreasing the percentage of cells in G0/G1 phase after 6 h of treatment (Figure 3A) and Figure S5A–E. The percentage of A375 cells in G2/M phase was 54.46 ± 2.94%, 51.43 ± 4.77% and 56.95 ± 3.09% for **S1**, **S19** and **S22**, respectively, compared to 33.64 ± 1.97% in control. These data were comparable with reference CA-4, where the percentage of arrested cells was 60.00 ± 2.54%. Surprisingly, after 24 h, when CA-4 induced mitotic block increased to 72.36 ± 1.10%, the percentage of G2/M phase cells treated with tested compounds was much lower and not significantly different from control. However, it should be pointed that simultaneously the number of hypodiploid DNA (sub-G0/G1) and/or polyploid DNA (population after G2/M phase) markedly increased (Figure 3B). As shown in Figure S5F–J, the sub-G0/G1 population increased significantly in **S1**, **S19** and **S22** treated cells which is considered as characteristic feature of cells undergoing apoptosis [29], whereas in CA-4 treated cells, a DNA index dramatically increased as a result of DNA polyploidization. Polyploidy is also typical for cells blocked in mitosis by colchicine (described as ‘‘c-mitosis’’ or ‘‘colchicine-mitosis’’), as a results of partial or complete absence of spindle apparatus following the breakdown of nuclear envelope, condensed chromosomes, and undivided centromeres [30]. Our data are consistent with Gilson et al. study, where after initial accumulation of H358 and HeLa cells in mitosis as a result of pyrrolopyrimidine (PP-13) treatment, the percentage of G2/M arrested cells was gradually decreasing, concomitantly with the accumulation in the sub-G1 and polyploid fraction [31]. As mentioned above, inhibition of tubulin polymerization and mitotic spindle formation may be one of the reasons for polyploid nuclei formation. Therefore, in the further study, the impact of **S1**, **S19** and **S22** compounds on mitotic spindle formation and tubulin polymerization was evaluated.

### 2.6. Impact on Mitotic Spindle Formation

As G2/M cell-cycle arrest is strongly correlated with microtubule disruption [32], in the next step the effect of tested compounds on mitotic spindle formation was evaluated using confocal microscopy imaging. After 6 h-incubation of A375 cells with **S1**, **S19** and **S22**, the cells were fixed and α-tubulin and chromosomes were stained. At first it should be emphasized that the morphological differences between treated and untreated cells were noticeable only during mitosis, confirming mitotic-specific activity of tested compounds. Similarly, spindle microtubules are more sensitive to colchicine than the interphase microtubules [33], whereas taxanes and vinca-alkaloids also affect interphase cells [34]. Cell-permeable small molecules that perturb mitosis without effects on microtubule/cytoskeleton in interphase may be useful in anticancer therapy and contribute to less pronounced adverse effects. For example, as differentiated neurons are in a post-mitotic state and are not be able to re-enter the cell cycle [35], this phase-specific activity may contribute to reduced neurotoxicity, which is one of the drawbacks of classical MTAs. 

**Figure 4:**
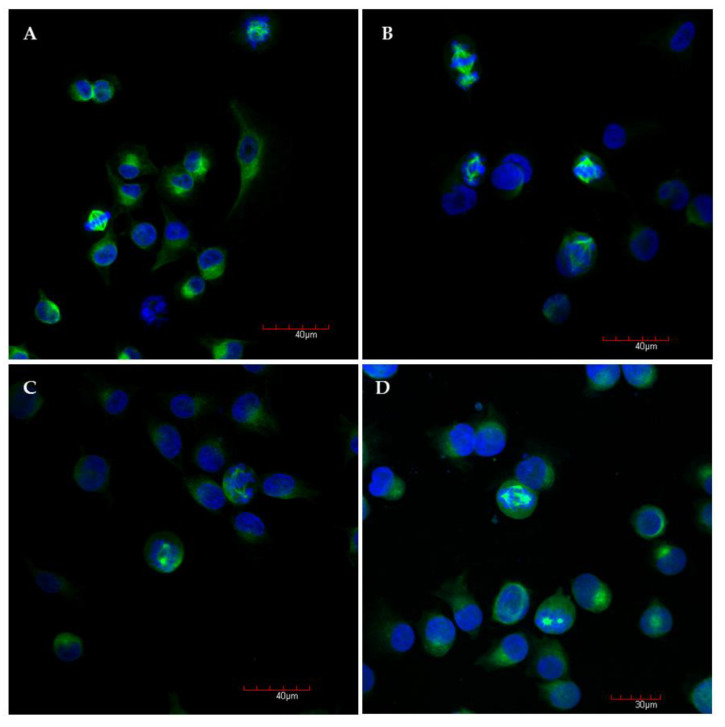
Representative confocal microscopy images of A375 cells after 6 h incubation with medium and 0.2% DMSO (**A**), compound **S1** (**B**), **S19** (**C**), **S22** (**D**) at concentration of 10 µM, with stained α-tubulin (green) and chromosomes (blue); magnification ×20 plus zoom ×3 during acquisition.

**Figure 5:**
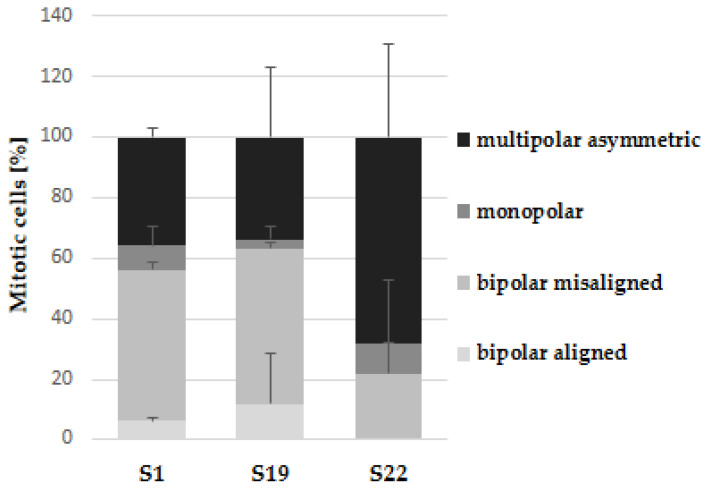
Quantification of mitotic spindle phenotypes of A375 cells after 6 h incubation with indicated compounds at 10 µM. Data are expressed as the mean and SD from two independent experiments.

As depicted in Figure 4A, the control cells in mitosis showed bipolar spindle formation with chromosomes arrangement in the central metaphase plate. In contrast, compound **S1**, **S19** and **S22** caused improper changes in mitotic spindle organization (Figure 4B–D). We could describe the three most common abnormal phenotypes: (1) bipolar spindles with chromosomes arrangement in the central metaphase and additional “cloud” of chromosomes around of centrioles (misalignment chromosomes), (2) monoastral spindles, (3) multipolar asymmetric asters (Figure 5). Observed aberrations were previously associated with strong defects in chromosome congression and alignment, and may be connected with prometaphase blockade [31]. Multipolar and “bipolar misaligned” spindles were described by Jaunky et al. in cells treated with colchicine and C75 (new CBSIs), depending on concentration and (in the case of C75) cell line used. The authors supposed that the discrepancy in mitotic phenotypes caused by C75 between different cell lines might be connected with genetically-dependent sensitivity to spindle disruption [36]. However, observed aberrations are not only specific for tubulin inhibitors but also other compounds targeting mitotic spindle. Namely, bipolar spindles with chromosomes aligned at metaphase and misaligned chromosomes located close to the spindle poles are also characteristic for inhibitors of kinesin-like protein (KIFC1) and centromere-associated protein E (CENP-E), kinesins essential for bipolar spindle formation and correct chromosome alignment, respectively [37,38]. Therefore, it is not possible to precisely define the molecular target of currently tested compounds on the base of observed phenotypes. 

**Figure 6:**
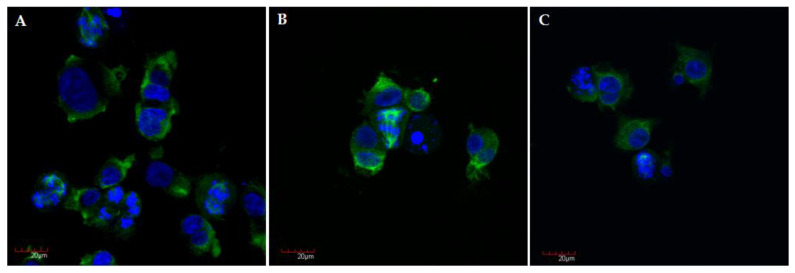
Representative confocal microscopy images of A375 cells after 24 h incubation with compound **S1** (**A**), **S19** (**B**), **S22** (**C**) at concentration of 10 µM, with stained α-tubulin (green) and chromosomes (blue); magnification ×40 plus zoom ×2 during acquisition.

In agreement with the flow cytometry analysis, the number of huge polynucleated cells and apoptotic bodies increased after 24 h of treatment (Figure 6). Observed changes are characteristic for mitotic cell death (MCD) often called a mitotic catastrophe. Typical features of this process include block in mitosis, mitotic spindle disorganization, failed chromosome segregation, and formation of multinucleated cells [39,40]. Mitotic catastrophe has been described as predominant form of cell death mediated by CA-4 and CA-4 analogue [41,42].

### 2.7. Tubulin Polymerization in Cells and Cell-Free Conditions

**Figure 7:**
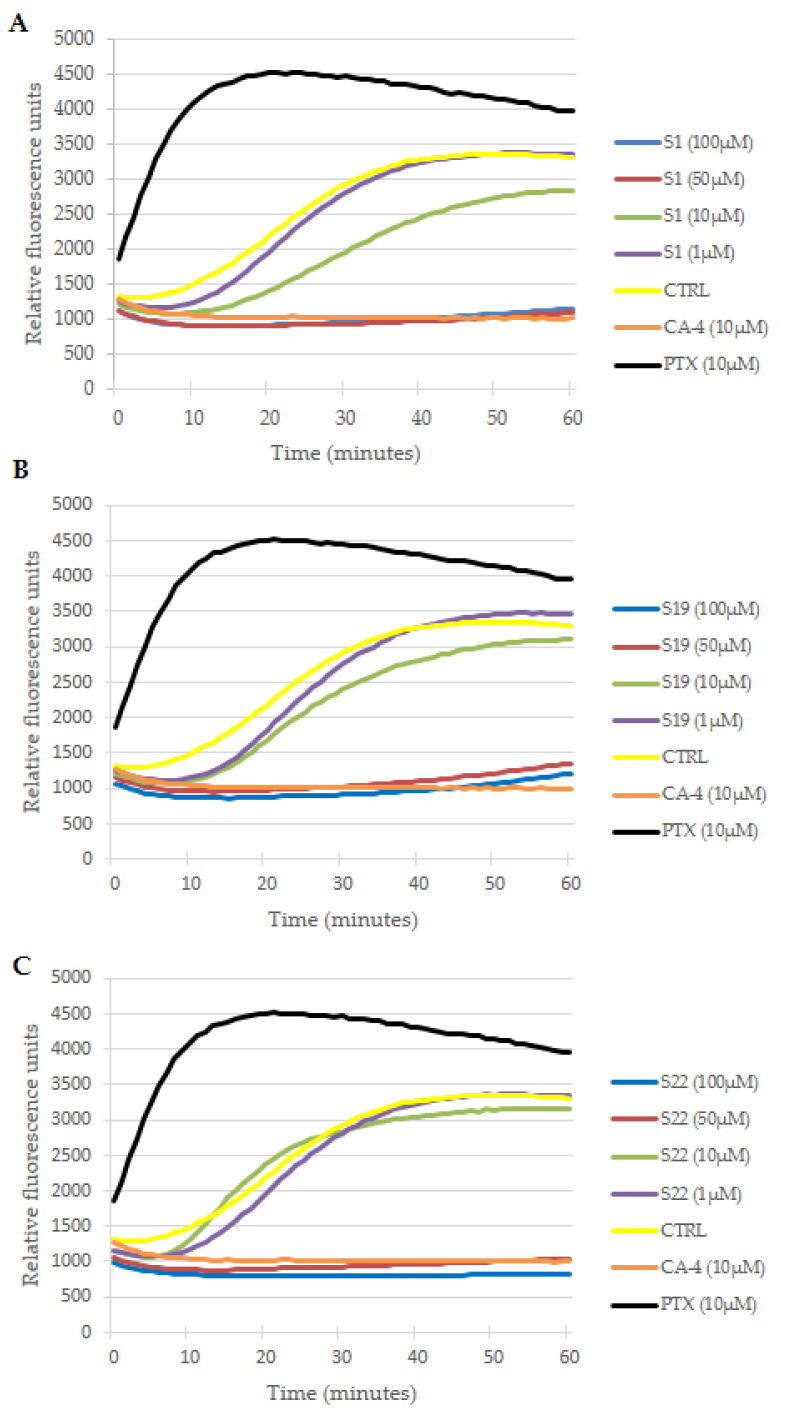
Effects of **S1** (**A**), **S19** (**B**) and **S22** (**C**) derivatives on tubulin polymerization in cell free conditions. Increasing relative fluorescence units is indicative of tubulin polymerization. Paclitaxel (PTX) and combretastatin (CA-4) were used as references and DMSO (0.2%) as a vehicle control. Curves of tested compounds represent the mean from at least three independent experiments. Error bars are not shown for the sake of clarity.

The observed aberrations in mitotic spindle morphology encouraged us to verify whether this effect was a result of interaction with microtubule dynamics, which is essential for a proper mitotic process [43]. A direct effect of **S1**, **S19** and **S22** derivatives on tubulin behavior in cell free conditions was evaluated using a fluorescence-based tubulin polymerization assay. The activity of the tested compounds was compared with paclitaxel (PTX), CA-4 and 0.2% DMSO as references and control, respectively. As shown in Figure 7, DMSO in the control sample had no direct effect on spontaneous tubulin self-assemble, where three typical phases of microtubule formation (namely: nucleation, growth and steady state inhibition) could be observed. The reference PTX (10 µM) eliminated the nucleation phase and enhanced the V max (maximal slope values for the growth phase), whereas CA-4 (10 µM) inhibited tubulin polymerization, resulting in a decrease in V max and reduction in final polymer mass protein. The overlapping curves of CA-4 (10 µM) and tested compounds at 50 µM and 100 µM indicated that **S1**, **S19** and **S22** act as tubulin polymerization inhibitors. However, inhibition of tubulin assembly by the tested compounds observed only at higher concentrations points that they are less active than reference drug in this regard. At 10 µM (green curves in Figure 7) **S1** was the most effective among the tested compounds, as delayed start of tubulin assembly, reduced microtubule polymerization speed and total polymer mass could still be clearly seen at that concentration. At 1 µM none of the investigated derivatives had any significant effect on tubulin polymerization (all purple curves finally overlapped with control in Figure 7). These observations were confirmed by IC_50_ evaluation presented in Table 3.

**Figure 8:**
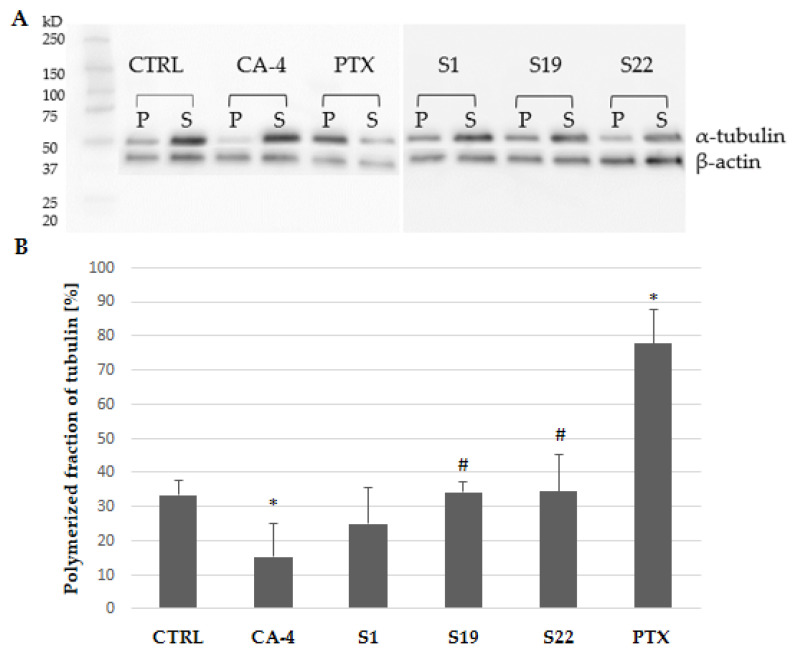
(**A**) Western blot analysis of polymerized microtubules (P) and soluble tubulin dimers (S) fraction in the cells after 6 h treatment with indicated compounds. Original images for blots in Supplementary Materials (Figure S6). (**B**) Percentage of α-tubulin in the pellet fraction after densitometric analysis of western blot gel. Each densitometric value of tubulin was normalized to the densitometric value of β-actin. Data are expressed as the mean and SD from four independent experiments; * *p* < 0.05 vs. ctrl; # *p* < 0.05 vs. CA-4).

The obtained data are in line with the results from another tubulin polymerization assay used (based on western blot method). Analysis was limited to the single concentration of the tested compounds (10 µM). As expected, CA-4 and PTX (used as references), increased the amount of tubulin in the soluble or polymeric fraction, respectively (Figure 8A). As shown in Figure 8B, α-tubulin polymer fraction was slightly but not significantly reduced in **S1**-treated A375 cells when compared with the control (24.84% vs. 33.36%). One could expect that the observed effect would be more distinct at higher concentrations, as our previously published data showed a reduction of polymerized tubulin fraction to 0.58% in **S1** exposed cells at 100 µM [9]. Compounds **S19** and **S22** did not influence tubulin polymerization as evaluated by a western blot method. 

### 2.8. Colchicine-Binding Site Study

The aforementioned common behavior of our compounds and colchicine encouraged us to verify the possible binding mode. For this purpose a molecular docking study was performed. Colchicine binds to tubulin at the interface between the α and β subunits. The majority of the compound is buried in the β subunit. As revealed by X-ray analysis of the tubulin-DAMA/colchicine complex structure, the A and C colchicine rings interact with the β-subunit of tubulin, while the B ring of the side chain interacts with the α-subunit. One of its methoxy oxygens of trimethoxyphenyl (TMP) moiety (A ring) is involved in hydrogen bonding with the thiol of Cys241β [2,45]. The Cys241β has been previously indicated as an important target for colchicine site agents [46]. According to Nguyen et al., all of the 15 structurally diverse CBSIs formed hydrogen bond with Cys241β (Cys239β in that study) [47]. Therefore, we focused on this crucial residue during molecular docking study. As shown in Figures S7 and S9 the B-ring of **S1** and **S22** (first possible mode of action) or A-ring (thiophene) of **S22** (Figure S10, second possible docking model) were directed toward Cys239β, but did not form hydrogen bond with sulphur atom of this amino acids. Bhattacharyya et al. indicated that the pi electron clouds of the thiophene and the phenyl rings of nocodazole (well known CBSIs) can act as hydrogen bond acceptors by forming a weak hydrogen bond coupled with hydrogen bond donor such as the thiol group of Cys239β [33]. However, recently data revealed that nocodazole did not interact with the α-tubulin and was situated deeper in β-tubulin binding pocket overlapping very little with colchicine in the regard of binding site [48]. Our findings confirm a crucial role of sulphur atom and -NH group of the pyridine-2(1*H*)thione (C-ring) moiety in hydrogen bond formation. Namely, the sulphur atom of **S1** showed hydrogen bonding interaction with amino acids of tubulin (Asn101α) (Figure S7). The **S19** formed two potential hydrogen bonds between sulphur atom and -NH group of compound and Gln247α and Ala354β, respectively (Figure S8). Whereas **S22** could form only one hydrogen bond between -S and Gln247β (Figure S9) or -NH and Val238β (Figure S10). Our data are in line with previous studies that pointed to the sulphur atom as an essential factor for anticancer activity of monastrol and other thio-derivaties (3,4-dihydropyrimidin-2(1*H*)-thiones). The substitution of sulphur by an oxygen led to the loss of the cytotoxic activity, probably due to the “soft nature” of the sulphur atom which makes compounds more nucleophilic [49].

**Figure 9:**
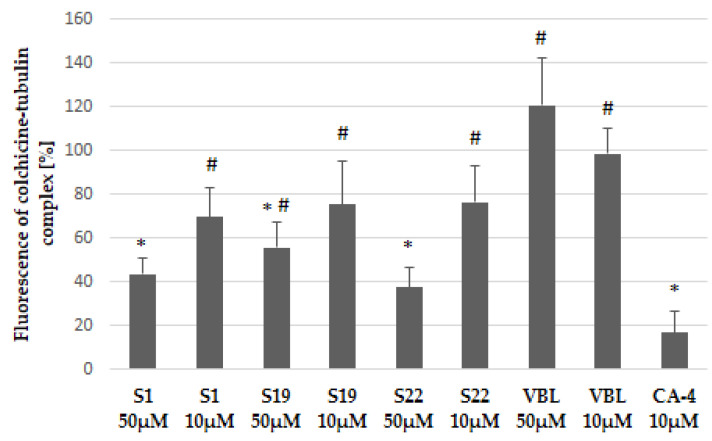
Colchicine competitive binding assay. Results have been shown as intrinsic fluorescence of colchicine-tubulin complex normalized to control (0.2% DMSO). CA-4 was used as a positive control whereas vinblastine (VBL), which binds at vinblastine site, was used as negative control. Data are expressed as the mean and SD from three independent experiments for **S1**, **S19**, **S22**, CA-4 and two independent experiments for VBL; * *p* < 0.05 vs. ctrl; # *p* < 0.05 vs. CA-4.

These molecular docking results were further supported by a fluorescence-based competitive binding assay. Namely, we evaluated the ability of compounds **S1**, **S19** and **S22** to compete with colchicine which intrinsic fluorescence increases upon binding to tubulin. We chose two the most critical concentration of tested compounds, selected during the cell free tubulin polymerization study (10 µM and 50 µM). As shown in Figure 9 the fluorescence of colchicine-tubulin complex was reduced in the presence of **S1**, **S19** and **S22** in dose-dependent manner suggesting that these compounds bind at the colchicine site and may belong to CBSIs. Vinblastine (VBL, used as negative control) did not compete with colchicine because it inhibits tubulin polymerization by binding at different site, located on β-tubulin chain. However, the investigated **S1**, **S19**, and **S22** compounds had lower affinity to colchicine-binding site than reference CA-4, and those findings were also in line with the results of the tubulin polymerization study described above.

## 3. Conclusions

The current study is a part of wider research related to an increasing interest in nitrogen- and sulphur-based heterocycles as promising anticancer agents [10]. A structure-activity relationship study resulted in identification and detailed characterization of 3,4-dihydropyridine-2(1*H*)-thione derivative with thiophene ring at C5 pyridine-2(1*H*)-thione (**S22**) with higher antiproliferative activity (IC_50_ equal 1.71 ± 0.58 µM) and better selectivity (SI = 21.09) against A375 cells than the starting compound **S1** (IC_50_ = 4.33 ± 1.00 µM, SI = 6.65). Moreover, all three the most active compounds from the antiproliferative study, namely **S1**, **S19** and **S22** showed better selectivity against A375 cancer cells than reference CA-4, suggesting their possible lower toxicity and wider therapeutic index. Additionally, mitotic-specific activity of the tested compounds (observed during cell cycle analysis and confocal microscopy imaging), might potentially contribute to decreased side effects compared with classical microtubule targeted agents (MTAs). Further study revealed that all the three tested compounds transiently arrested cells in mitosis, leading to apoptotic cell death. Moreover, we proved that compounds **S1**, **S19** and **S22** disrupted mitotic spindle organization and inhibited tubulin polymerization via colchicine binding site in dose-dependent manner. However, direct activity against tubulin was not as high as could be expected from results of cell-based study. Hence, obtained findings may suggest more complexed mechanism of action. The higher anticancer selectivity of **S22** compared with the reference microtubule inhibitor (CA-4) may be also connected with multitarget mode of action, including not only microtubules but also kinesins and kinases involved in mitosis and overexpressed in cancer cells [50,51]. The cooperation of kinesins and kinases in microtubule assembly is well documented and pointed as a new opportunity to design potential multitarget antimitotic inhibitors [52]. For example nocodazol, a colchicine binding site agent was found to simultaneously inhibit various cancer-related kinases, including ABL1, c-KIT, BRAF, MEK1 (MAP2K1), MEK2 (MAP2K2), and MET [53]. Frezzato et al., demonstrated that a remarkable ability of nocodazole to discriminate between leukaemic and normal cells was connected with inhibition of Src-kinase Lyn, overexpressed and crucially involved in the neoplastic B-cell survival [54]. We supposed that anticancer selectivity of compound **S22** may be also connected with the fact that distribution of β-tubulin isotypes varies among different tumor and normal cells, which modulates their sensitivity to chemotherapeutic drugs [55,56]. However, both hypothesis should be verified in a further study.

## 4. Materials and Methods

### 4.1. Experimental Part

Melting points were determined on a Boetius hot stage apparatus. ^1^H and ^13^C NMR spectroscopic measurements were performed on a Bruker DPX 400 Avance III HD spectrometer (Billerica, MA, USA, operating at 400.2 and 100.6 MHz, respectively. TMS was used internal standard, δ_H,C_ = 0 ppm). For NMR analyses MestReNova (version: 12.0.4 Santiago de Compostela, Spain) program was used. For detailed peak assignments, 2D spectra were acquired using Bruker software (^1^H,^1^H DFQCOSY; ^13^C,^1^H COSY; ^1^H,^13^C HMBC). The ^1^H,^13^C HMBC long-range correlations were acquired for *J*_C,H_ = 10 Hz. The standard abbreviation for multiplicities were used (s = singlet, d =doublet, t = triplet, q =quartet, quint = quintet, m = multiplet, sxt = sextet, spt = septet, etc. Gas chromatography-mass spectrometry (GC-MS) measurements were carried out on a Hewlett-Packard instrument model HP 6890 equipped with a mass detector HP 5973 and on an Agilent 7820A GC system equipped with a mass (Agilent 5977E MSD) detector. HRMS analyses (ESI+) were performed on a Waters LCT premier XE (TOF) using acetonitrile as solvent. Elemental analyses were carried out on Thermo Scientifc™ FLASH 2000 CHNS/O Analyzer (Waltham, MA, USA). *n*-BuLi (2.5 M in hexane), *sec*-BuLi (1.4 M in cyclohexane), MeLi (3.1 M in diethoxymethane), BnMgCl (2.0 M in THF) were purchased from Sigma-Aldrich (Merck Group, St. Louis, MO, USA). Anhydrous toluene and THF were purified by distillation over sodium metal under argon prior to use. Products were purified by flash column chromatography on silica gel (63–200 μm, Merck, Darmstadt, Germany) using appropriate solvents.

### 4.2. Synthesis of Compounds **S1**-**S22**

General procedure for the synthesis of 5-aryl-4-benzylpyridine-2(1*H*)-thiones **S1**-**S22** by the oxygen–sulphur exchange:

To a solution of aryl- and benzyl-substituted pyridin-2(1*H*)-one (0.308 g, 1.2 mmol) in anhydrous toluene (40 mL) Lawesson reagent (Fluorchem, Derbyshire, United Kingdom; 0.27 g, 0.66 mmol) was added and the mixture was stirred for 4 h at 80 °C (oil bath temperature). After this time the mixture was cooled to room temperature and most of the solvent (2/3 volume) was distilled off in vacuo and the remaining mixture was twice chromatographed on silica gel. The obtained solids were crystallized from appropriate solvents.

### 4.3. Purification Conditions and Spectroscopic Data

(4*RS*)-4-Benzyl-5-phenyl-3,4-dihydropyridine-2(1*H*)-thione [9] (**S1**): The crude product purified by column chromatography on silica gel using a mixture of ethyl acetate and *n*-hexane yielded pale yellow solid in 68%. Mp. 143–144 °C (*n*-hexane: ethyl acetate). ^1^H NMR (400 MHz, CDCl_3_) δ 2.58 (dd, *J* = 13.6, 10.9 Hz, 1 H, 4-CHH), 2.79 (dd, *J* = 17.3, 7.1 Hz, 1 H, CHH-3), 2.83 (dd, *J* = 13.6, 3.7 Hz, 1 H, 4-CHH), 2.98–3.05 (m, 1 H, CH-4), 3.21 (d, *J* = 17.3 Hz, 1 H, CHH-3), 6.56 (d, *J* = 4.6 Hz, 1 H, =CH-6), 7.21–7.48 (m, 10 H, 2× C_6_H_5_), 9.66 (br. s., 1 H, NH). ^13^C NMR (100.6 MHz CDCl_3_): δ 35.73 (CH-4), 37.11 (4-CH_2_), 41.68 (CH_2_-3), 120.13 (=CH-6), 124.95, 126.53 (C_6_H_5_), 126.99 (C-5), 127.71, 128.50, 128.98, 129.55, 136.12, 138.48 (C_6_H_5_), 197.96 (C=S). GC-MS (EI, 70 eV): *m/z* = 279 (36), [M^+^], 188 (100), 154 (19), 128 (30), 91 (15). HRMS (ESI-TOF): calcd. for C_18_H_18_NS 280.1160 [M + H]^+^; found 280.1166.

(4*RS*)-4-Benzyl-1-methyl-5-phenyl-3,4-dihydropyridine-2(1*H*)-thione (**S2**): The crude product purified twice by column chromatography (the first column: SiO_2_, *n*-hexane: ethyl acetate, 9: 1, the second column: SiO_2,_ chloroform), gave yellow solid in 96% yield. Mp. 127–129 °C (petroleum ether: ethyl acetate). ^1^H NMR (400 MHz, CDCl_3_) δ 2.53 (dd, *J* = 13.5, 10.3 Hz, 1H, 4-CHH), 2.79 (dd, *J* = 13.5, 3.6 Hz, 1H, 4-CHH), 2.88 (ddq, *J* = 16.3, 6.5 Hz, 0.9 1H, CHH-3), 2.95 (dddd, 10.3, 6.5, 3.6, 1.5, 1H, CH-4), 3.34 (dd, *J* = 16.3, 1.5 Hz, 1H, CHH-3), 3.59 (d, *J* = 0.9 Hz, 3H, NCH_3_), 6.64 (s, 1H, =CH-6), 7.16–7.26 (m, 3H, C_6_H_5_), 7.26–7.34 (m, 3H, C_6_H_5_), 7.35–7.43 (m, 2H, C_6_H_5_), 7.43–7.48 (m, 2H, C_6_H_5_). ^13^C NMR (101 MHz CDCl_3_) *δ* 35.86 (CH-4), 37.29 (4-CH_2_), 42.27 (N-CH_3_), 44.19 (CH_2_-3), 125.07 (C_6_H_5_), 126.40 (=CH-6), 126.49, 127.75 (C_6_H_5_), 128.18 (=C-5), 128.39 128.99, 129.61, 136.18, 138.59 (C_6_H_5_), 195.72 (C=S). GC-MS (EI, 70 eV): *m/z* = 293 (19), [M^+^], 204 (6), 203 (15), 202 (100), 201 (7), 186 (4), 170 (4), 168 (5), 128 (14), 127 (5), 115 (7), 91(9). HRMS (ESI-TOF): *m*/*z* [M^+^+H] calcd. for C_19_H_20_NS 294.1316; found: 294.1323.

(4*RS*)-4-Benzyl-5-phenyl-1-propyl-3,4-dihydropyridine-2(1*H*)-thione (**S3**): The crude product purified twice by column chromatography (the first column: SiO_2_, *n*-hexane: ethyl acetate, 10: 1, the second column: SiO_2,_ chloroform), gave yellow solid in 89% yield. Mp. 105–107 °C (petroleum ether: ethyl acetate). ^1^H NMR (400 MHz, CDCl_3_) δ 1.02 (t, *J* = 7.4 Hz, 3H, N-CH_3_), 1.72–1.89 (m, 2H, CH_2_) 2.53 (dd, *J* = 13.5, 10.3 Hz, 1H, 4-CHH), 2.78 (dd, *J* = 13.5, 3.6 Hz, 1H, 4-CHH), 2.86 (dd, *J* = 16.2, 6.6 Hz, 1H, CHH-3), 2.89–3.00 (m, 1H, CH-4), 3.34 (dd, *J* = 16.2, 1.5, 1H, CHH-3), 3.83 (ddd, *J* = 13.1, 8.7, 6.5 Hz, 1H, NCHH), 4.38 (ddd, *J* = 13.0, 8.8, 6.4 Hz, 1H, NCHH), 6.63 (s, 1H, =CH-6), 7.14–7.34 (m, 6H, C_6_H_5_), 7.37–7.48 (m, 4H, C_6_H_5_). ^13^C NMR (101 MHz CDCl_3_): *δ* 11.36 (CH_3_), 20.57 (CH_2_), 35.76 (CH-4), 37.03 (4-CH_2_), 44.55 (CH_2_-3), 55.24 (NCH_2_), 125.06 (C_6_H_5_), 125.47(=CH-6), 126.46, 127.69 (C_6_H_5_), 128.36 (=C-5), 128.41, 128.95, 129.60, 136.31, 138.64 (C_6_H_5_), 195.29 (C=S). GC-MS (EI, 70 eV): *m/z* = 321 (27), [M^+^], 230 (100), 188 (44), 154 (15), 128 (19), 91 (16), 65 (5). HRMS (ESI-TOF): *m*/*z* calcd for C_21_H_24_NS [M+H]^+^, 322.1629; found: 322.1635.

(4*RS*)-1,4-Dibenzyl-5-phenyl-3,4-dihydropyridine-2(1*H*)-thione (**S4**): The crude product purified twice by column chromatography (the first column: SiO_2_, *n*-hexane: ethyl acetate, 9: 1, the second column: SiO_2,_ chloroform), gave yellow solid in 85% yield. Mp. 73–76 °C (petroleum ether: ethyl acetate). ^1^H NMR (400 MHz, CDCl_3_): δ 2.54 (dd, *J* = 13.6, 9.8 Hz, 1H, 4-CHH), 2.77 (dd, *J* = 13.6, 3.5 Hz, 1H, 4-CHH), 2.92–2.99 (m, 2H, CHH-3, CH-4), 3.41–3.48 (m, 1H, CHH-3), 5.39 (d, *J* = 14.8 Hz, 1H, NCHH), 5.53 (d, *J* = 14.8 Hz, 1H, NCHH), 6.64 (s, 1H, =CH-6), 7.20–7.45 (m, 15H, C_6_H_5_). ^13^C NMR (101 MHz CDCl_3_): δ 35.91 (CH-4), 37.02 (4-CH_2_), 44.56 (CH_2_-3), 55.88 (NCH_2_), 124.77 (=CH-6), 125.10, 126.49, 127.72, 127.78, 127.96, 128.43 (C_6_H_5_), 128.71 (=C-5), 128.85, 128.88, 129.58, 135.70, 136.16, 138.61 (C_6_H_5_), 196.49 (C=S). GC-MS (EI, 70 eV): *m/z* = 369 (11), [M^+^], 279 (8), 278 (40), 123 (5), 92 (8), 91 (100), 65 (7). HRMS (ESI-TOF): *m*/*z* calcd for C_25_H_24_NS [M+H]^+^, 370.1629; found: 370.1633.

(6*RS*)-6-Benzyl-5-phenyl-3,6-dihydropyridine-2(1*H*)-thione (**S6**): The crude product purified twice by column chromatography (the first column: SiO_2_, *n*-hexane: ethyl acetate, 6: 1, the second column: SiO_2,_ chloroform), gave pale yellow solid in 90% yield. Mp. 137–138 °C (petroleum ether: ethyl acetate). ^1^H NMR (400 MHz, CDCl_3_): δ 2.72 (dd, *J* = 13.8, 6.4 Hz 1H, 6-CHH), 2.79 (dt, *J* = 23.2, 3.1 Hz, 1H, CHH-3), 3.07 (dd, *J* = 13.8, 3.8 Hz, 1H, 6-CHH), 3.48 (ddd, *J* = 23.2, 5.1, 3.1 Hz, 1H, CHH-3), 4.86 (ddt, *J* = 6.4, 3.1, 3.8 Hz, 1H, CH-6), 5.90 (dd, *J* = 5.1, 3.1 Hz, 1H, =CH-4), 7.00–7.14 (m, 2H, C_6_H_5_), 7.24–7.34 (m, 3H, C_6_H_5_), 7.34–7.53 (m, 5H, C_6_H_5_), 9.26 (br. s, 1H, NH). ^13^C NMR (101 MHz CDCl_3_): δ 40.00 (CH_2_-3), 40.83 (6-CH_2_), 58.3 (CH-6), 120.60 (=CH-4), 126.00, 127.23, 128.55, 129.03, 130.05 (C_6_H_5_), 134.27 (=C-5), 135.22, 137.27 (C_6_H_5_), 199.5 (C=S). GC-MS (EI, 70 eV): *m/z* = 279 (27), [M^+^], 188 (100), 154 (19), 128 (29), 91 (16). HRMS (ESI-TOF): *m*/*z* calcd for C_18_H_18_NS [M+H]^+^, 280.1160; found: 280.1161.

(6*RS*)-6-Benzyl-1-methyl-5-phenyl-3,6-dihydropyridine-2(1*H*)-thione (**S7**): The crude product purified twice by column chromatography (the first column: SiO_2_, *n*-hexane: ethyl acetate, 6: 1, the second column: SiO_2,_ chloroform), gave dark red oil in 78% yield. ^1^H NMR (400 MHz, CDCl_3_): δ 2.22 (ddd, *J* = 22.6, 3.5, 2.2 Hz, 1H, CHH-3), 2.78 (dd, *J* = 13.9, 4.1 Hz, 1H, 6-CHH), 3.18 (dd, *J* = 13.9, 4.5 Hz, 1H, 6-CHH), 3.51 (ddd, *J* = 22.4, 5.7, 1.9 Hz, 1H, CHH-3), 3.62 (d, *J* = 0.8 Hz, 3H, N-CH_3_), 4.92 (dddd, *J* = 4.5, 4.1, 3.4, 1.9 Hz, 1H, CH-6), 5.9 (dd, *J* = 5.7, 2.2 Hz, 1H, =CH-4), 6.78–7.06 (m, 2H, C_6_H_5_), 7.12–7.31 (m, 3H, C_6_H_5_), 7.32–7.48 (m, 5H, C_6_H_5_). ^13^C NMR (101 MHz CDCl_3_): δ 37.64 (6-CH_2_), 42.46 (CH_2_-3), 42.73 (NCH_3_), 66.24 (CH-6), 121.50 (=CH-4), 125.85, 127.39, 128.22, 128.29, 129.04, 130.13 (C_6_H_5_), 134.66 (=C-5), 134.73, 137.06 (C_6_H_5_), 197.40 (C=S). GC-MS (EI, 70 eV): *m/z* = 293 (22), [M^+^], 202 (100), 170 (6), 128 (11), 91 (8), 65 (63), 42 (8). HRMS (ESI-TOF): *m*/*z* calcd for C_19_H_20_NS [M+H]^+^, 294,1316; found: 294,1314.

(4*RS*)-4-Benzyl-5-(4-fluorophenyl)-3,4-dihydropyridine-2(1*H*)-thione (**S8**): The crude product purified twice by column chromatography (the first column: SiO_2_, *n*-hexane: ethyl acetate, 10: 1, the second column: SiO_2,_ chloroform), gave yellow solid in 90% yield. Mp. 144–147 °C (hexane: ethyl acetate). ^1^H NMR (400 MHz, CDCl_3_): δ 2.57 (dd, *J* = 13.7, 10.7 Hz, 1H, 4-CHH), 2.73–2.84 (m, 2H, 4-CHH_,_ CHH-3), 2.96 (dddd, *J* = 10.7, 6.2, 4.0, 1.8 Hz, 1H, CH-4), 3.20 (dd, *J* = 17.2, 1.5 Hz, 1H, CHH-3), 6.50 (d, *J* = 4.6 Hz, 1H, =CH-6), 7.02–7.12 (m, 2H, C_6_H_5_), 7.15–7.46 (m, 7H, C_6_H_5_), 9.95 (br. s, 1H, NH). ^13^C NMR (101 MHz CDCl_3_): δ 35.99 (CH-4), 37.16 (4-CH_2_), 41.85 (CH_2_-3), 115.89 (d,^2^*J*_C-F_ = 21.6 Hz, CH-3′, CH-5′), 120.05 (=CH-6), 126.22 (=C-5), 126.59 (C_6_H_5_), 126.73 (d,^3^ *J*_C-F_ = 8.0 Hz, CH-2′, CH-6′), 128.50 (C_6_H_5_), 129.50 (C_6_H_5_), 132.40 (d, ^4^*J*_C-F_ = 3.4 Hz, C-1′), 138.32 (C_6_H_5_), 162.24 (d, ^1^*J*_C-F_ = 247.9 Hz, C-4′), 197.71 (C=S). GC-MS (EI, 70 eV): *m/z* = 279 (37) [M^+^], 207(14), 206 (100), 173 (9), 172 (21), 146 (30), 91 (17). HRMS (ESI-TOF): *m*/*z* calcd for C_18_H_17_FNS [M+H]^+^, 298.1066; found: 298.1064.

(4*RS*)-4-Benzyl-5-(3,4-difluorophenyl)-3,4-dihydropyridine-2(1*H*)-thione (**S9**): The crude product purified twice by column chromatography (the first column: SiO_2_, *n*-hexane: ethyl acetate, 8: 1, the second column: SiO_2,_ chloroform), gave yellow solid in 95% yield. Mp. 142–149 °C (hexan: ethyl acetate). ^1^H NMR (400 MHz, CDCl_3_): δ = 2.58 (dd, *J* = 13.6, 10.5 Hz, 1H, 4-CHH), 2.73–2.84 (m, 2H, CHH-3, 4-CHH), 2.92 (dddd, *J* = 10.5, 6.4, 4.2, 1.5, 1H, CH-4), 3.22 (dd, *J* = 16.8, 1.5 Hz, 1H, CHH-3), 6.51 (d, *J* = 4.6 Hz, 1H, =CH-6), 7.00–7.46 (m, 8H, ArH), 9.76 (s, 1H, NH). ^13^C NMR (101 MHz CDCl_3_): δ 35.93 (CH-4), 37.29 (4-CH_2_), 41.99 (CH_2_-3), 114.02 (d, ^2^*J* = 18.2 Hz, CH-2′), 117.75 (d, ^2^*J* = 17.4 Hz, CH-5′), 120.85 (d, ^5^*J* = 1.5 Hz, =CH-6), 121.06 (dd, *J* = 6.1, 3.4 Hz, CH-6′), 125.11 (=C-5), 126.73, 128.58, 129.51 (C_6_H_5_), 133.62 (dd, *J* = 5.9, 4.3 Hz, C-1′), 138.11 (C_6_H_5_), 148.41 (dd, *J* = 250.7, 12.6 Hz, C-3′), 150.68 (dd, *J* = 248.9, 12.5 Hz, C-4′), 198.1 (C=S). GC/MS (EI, 70 eV): *m/z* = 316 (6) [M^+^], 315 (27), [M^+^], 225 (14), 224 (100), 191 (8), 190 (20), 164 (25), 163 (6), 91 (21), 65 (7). HRMS (ESI-TOF): *m*/*z* calcd for C_18_H_16_F_2_NS [M+H]^+^, 316.0972; found: 316.0981.

(4*RS*)-4-Benzyl-5-(4-chlorophenyl)-3,4-dihydropyridine-2(1*H*)-thione (**S10**): The crude product purified twice by column chromatography (the first column: SiO_2_, *n*-hexane: ethyl acetate, 3: 1, the second column: SiO_2,_ chloroform), gave yellow solid in 78% yield. Mp. 161–163 °C (hexane: ethyl acetate). ^1^H NMR (400 MHz, CDCl_3_): δ 2.57 (dd, *J* = 13.6, 10.7 Hz, 1H, 4-CHH), 2.73–2.84 (m, 2H, 4-CHH, CHH-3), 2.97 (dddd, *J* = 10.7, 6.1, 4.0, 1.8 Hz, 1H, CH-4), 3.21 (ddd, *J* = 17.1, 1.8, 0.9 Hz, 1H, CHH-3), 6.55 (d, *J* = 4.6 Hz, 1H, =CH-6), 7.13–7.44 (m, 9H, C_6_H_5,_ C_6_H_4_), 9.53 (br. s, 1H, NH). ^13^C NMR (101 MHz CDCl_3_): δ 35.78 (CH-4), 37.21 (4-CH_2_), 41.82 (CH_2_-3), 120.45 (=CH-6), 125.83 (=C-5), 126.22, 126.64, 128.54, 129.11, 129.51, 133.38, 134.73, 138.25 (C_6_H_5,_ C_6_H_4_), 198.12 (C=S). GC-MS (EI, 70 eV): *m/z* = 313 (31) [M^+^], 225 (5), 224 (37), 222 (100), 221 (12), 188 (16), 162 (17), 128 (9), 115 (10), 91 (23), 65 (8). HRMS (ESI-TOF): *m*/*z* calcd for C_18_H_17_ClNS [M+H]^+^, 314.0770; found: 314.0782.

(4*RS*)-4-Benzyl-5-(3-methoxyphenyl)-3,4-dihydropyridine-2(1*H*)-thione (**S11**): The crude product purified twice by column chromatography (the first column: SiO_2_, *n*-hexane: ethyl acetate, 6: 1, the second column: SiO_2,_ chloroform), gave yellow solid in 96% yield. Mp. 137–139 °C (*n*-hexane: ethyl acetate). ^1^H NMR NMR (400 MHz, CDCl_3_): δ 2.57 (dd, *J* = 13.6, 11.0 Hz, 1H, 4-CHH), 2.78 (dd, *J* = 17.0, 6.7 Hz, 1H, CHH-3), 2.83 (dd, *J* = 13.6, 3.7 Hz, 1H, 4-CHH), 2.99 (dddd *J* = 11.0, 6.7, 3.7, 1.8 Hz 1H, CH-4), 3.20 (ddd, *J* = 17.0, 1.8, 1.0 Hz, 1H, CHH-3), 3.85 (s, 3H, OCH_3_), 6.56 (d, *J* = 4.6 Hz, 1H, =CH-6), 6.85 (ddd, *J* = 8.2, 2.5, 0.8 Hz, 1H, C_6_H_4_), 6.97 (t, *J* = 2.1 Hz, 1H, C_6_H_4_), 7.05 (ddd, *J* = 7.8, 1.8, 0.9 Hz, 1H, C_6_H_4_), 7.16–7.39 (m, 6H, C_6_H_5,_ C_6_H_4_), 9.69 (br. d, *J* = 4.6 Hz, 1H); ^13^C NMR (100 MHz, CDCl_3_): δ 35.83 (CH-4), 37.17 (4-CH_2_), 41.70 (CH_2_-3), 55.33 (OCH_3_), 111.10, 112.73, 117.49 (C_6_H_4_), 120.39 (=CH-6),126.56 (C_6_H_5_), 126.84 (=C-5), 128.52, 129.57, 129.98, 137.67, 138.50, 160.06 (C_6_H_5,_ C_6_H_4_), 198.07 (C=S). GC-MS (EI, 70 eV): *m/z* = 309 (30) [M+], 219 (15), 218 (100), 203 (6), 185 (7), 158 (14), 115 (11), 91 (17). HRMS (ESI-TOF): *m*/*z* calcd for C_19_H_20_NOS [M+H]^+^, 310.1266; found: 310.1252.

(4*RS*)-4-Benzyl-5-(3,4-dimethoxyphenyl)-3,4-dihydropyridine-2(1*H*)-thione (**S12**): The crude product purified twice by column chromatography (the first column: SiO_2_, *n*-hexane: ethyl acetate, 3: 1, the second column: SiO_2,_ chloroform), gave yellow solid in 88% yield. Mp. 135–138 °C. ^1^H NMR (400 MHz, CDCl_3_): δ 2.58 (dd, *J* = 13.6, 10.8 Hz, 1H, 4-CHH), 2.73–2.85 (m, 2H, 4-CHH_,_ CHH-3), 2.97 (dddd, *J* = 10.8, 6.4, 3.8, 1.8 Hz, 1H, CH-4), 3.21 (ddd, *J* = 17.1, 1.8, 1.0 Hz, 1H, CHH-3), 3.92 (s, 3H, OCH_3_), 3.92 (s, 3H, OCH_3_), 6.50 (d, *J* = 4.5 Hz, 1H, CH-6), 6.87–6.93 (m, 2H, C_6_H_3_), 7.03 (dd, *J* = 8.3, 2.2 Hz, 1H, C_6_H_3_), 7.19–7.34 (m, 5H, C_6_H_5_), 9.76 (br. d, *J* = 4.5 Hz, 1H, NH). ^13^C NMR (100 MHz, CDCl_3_): δ 36.08 (CH-4), 37.24 (4-CH_2_), 41.78 (CH_2_-3), 55.99 (two OCH_3_), 108.28, 111.44, 117.79 (C_6_H_3_), 119.11 (=CH-6), 126.57 (C_6_H_5_), 127.18 (=C-5), 128.54, 129.01, 129.53, 138.65, 148.95, 149.26 (C_6_H_3_, C_6_H_5_), 197.42 (C=S). GC-MS (EI, 70 eV): *m/z* = 339 (64) [M+], 249 (16), 248 (100), 233 (7), 232 (18), 215 (77), 200 (7), 188 (13), 172 (6), 115 (8), 91 (31), 77 (4), 65 (8). HRMS (ESI-TOF): *m*/*z* calcd for C_20_H_21_NNaO_2_S [M+Na]^+^ 362.1191; found: 362.1197.

(4*RS*)-4-Benzyl-5-(4-fluorophenyl)-1-methyl-3,4-dihydropyridine-2(1*H*)-thione (**S13**): The crude product purified twice by column chromatography the first column (SiO_2_, *n*-hexane: ethyl acetate, 8: 1, then chloroform), gave yellow solid in 89% yield. Mp. 143–147 °C. ^1^H NMR (400 MHz, CDCl_3_): δ 2.53(dd, *J* = 13.7, 9.5 Hz, 1H, 4-CHH), 2.74 (dd, *J* = 13.7, 3.6 Hz, 1H, 4-CHH), 2.84–2.93 (m, 2H, CH-4, CHH-3), 3.29–3.39 (m, 1H, CHH-3), 3.59 (s, 3H, N-CH_3_), 6.57 (s, 1H, CH-6), 7.04–7.12 (m, 2H, C_6_H_5_), 7.16–7.24 (m, 3H, C_6_H_5_), 7.25– 7.30 (m, 2H), 7.35–7.44 (m, 2H, C_6_H_5_). ^13^C NMR (100 MHz, CDCl_3_): δ 36.13 (CH-4), 37.31 (4-CH_2_), 42.25 (N-CH_3_), 44.28 (CH_2_-3), 115.9 (d, ^2^*J*_C-F_ = 21,8 Hz, CH-3′, CH-5′), 126.28 (d, ^6^*J* = 1.9 Hz, =CH-6), 126.54 (C_6_H_5_), 126.83 (d, ^3^*J*_C-F_ = 8.0 Hz, CH-2′, CH-6′), 127.36 (=C-5), 128.39 (2C), 129.55 (2C, C_6_H_5_), 132.40 (d, ^4^*J*_C-F_ = 3.3 Hz, C-1′), 138.42 (C_6_H_5_), 162.28 (d, ^1^*J*_C-F_ = 248.0 Hz, C-4′), 195.56 (C=S). GC/MS GC-MS (EI, 70 eV): *m/z* = 311 (20) [M+], 220 (100), 146 (15), 91 (44). HRMS (ESI-TOF): *m*/*z* calcd for C_19_H_19_FNS [M+H]^+^, 312.1222; found: 312.1216. 

(4*RS*)-4-Benzyl-5-(3,4-difluorophenyl)-1-methyl-3,4-dihydropyridine-2(1*H*)-thione (**S14**): The crude product purified twice by column chromatography the first (SiO_2_, *n*-hexane: ethyl acetate, 10: 1, then chloroform), gave yellow solid in 96% yield. Mp. 128–132 °C. ^1^H NMR (400 MHz, CDCl_3_): δ 2.55 (dd, *J* = 13.4, 9.7 Hz, 1H, 4-CHH), 2.73 (dd, *J* = 13.4, 3.9 Hz, 1H, 4-CHH), 2.81–2.87 (m, 1H, CH-4), 2.90 (dd, *J* = 15.1, 6.6 Hz, 1H, CHH-3), 3.36 (d, *J* = 15.1 Hz, 1H, CHH-3), 3.59 (s, 3H, NCH_3_), 6.59 (s, 1H, =CH-6), 7.07–7.33 (m, 8H, C_6_H_5_). ^13^C NMR (100 MHz, CDCl_3_): δ 36.03 (CH-4), 37.43 (4-CH_2_), 42.29 (N-CH_3_), 44.40 (CH_2_-3), 114.09 (d, ^2^*J* = 18.2 Hz, CH-2′), 117.74 (d, ^2^*J* = 17.5 Hz, CH-5′), 121.13 (dd, *J* = 6.2, 3.3 Hz, CH-6′), 126.20 (=C-5), 126.66 (C_6_H_5_), 127.02 (=CH-6), 128.45, 129.53 (C_6_H_5_), 133.62 (dd, *J* = 6.2, 3.8 Hz, C-1′), 138.21 (C_6_H_5_), 149.66 (dd, *J* = 249.9, 13.8 Hz, C-3′), 150.53 (dd, *J* = 248.6, 13.2 Hz, C-2′), 195.97 (C=S). GC-MS (EI, 70 eV): *m/z* = 329 (23) [M+], 238 (100), 164 (10). HRMS (ESI-TOF): *m*/*z* calcd. for C_19_H_17_F_2_NNaS [M+Na]^+^ 352.0947; found: 352.0946.

(4*RS*)-4-Benzyl-5-(4-chlorophenyl)-1-methyl-3,4-dihydropyridine-2(1*H*)-thione (**S15**): The crude product purified twice by column chromatography (the first column: SiO_2_, *n*-hexane: ethyl acetate, 10: 1, the second column SiO_2_, chloroform), gave yellow solid in 84% yield. Mp. 154–156 °C. ^1^H NMR (400 MHz, CDCl_3_): δ 2.53 (dd, *J* = 13.5, 9.8 Hz, 1H, 4-CHH), 2.67–2.79 (m, 1H, CHH-3), 2.84–3.00 (m, 2H, CH-4, 4-CHH), 3.22–3.43 (m, 1H, CHH-3), 3.58 (s, 3H, NCH_3_), 6.62 (s, 1H, =CH-6), 7.17–7.23 (m, 3H, C_6_H_5_), 7.25–7.31 (m, 2H, C_6_H_5_), 7.35 (s, 4H, C_6_H_4_). ^13^C NMR (100 MHz, CDCl_3_): δ 35.84 (CH-4), 37.35 (4-CH_2_), 42.27 (NCH_3_), 44.27 (CH_2_-3), 126.31, 126.57 (ArH), 126.66 (=CH-6), 126.95 (=C-5), 128.40, 129.10 (ArH), 129.54 (ArH), 133.39, 134.75, 138.34 (Ar)_,_ 195.82 (C=S). GC-MS (EI, 70 eV): *m/z* = 327 (23) [M+], 239 (5), 238 (38), 236 (100), 235 (7), 201 (6), 128 (6), 115 (6), 91 (10), 65 (5). HRMS (ESI-TOF): *m*/*z* calcd. for C_19_H_18_ClNNaS [M+Na]^+^ 350.0746; found: 350.0755.

(4-*RS*)-4-Benzyl-5-(3-methoxyphenyl)-1-methyl-3,4-dihydropyridine-2(1*H*)-thione (**S16**): The crude product purified twice by column chromatography (the first column: SiO_2_, *n*-hexane: ethyl acetate, 10: 1, the second column: SiO_2_, chloroform), gave yellow solid in 98% yield. Mp. 106–109 °C (hexane: ethyl acetate). ^1^H NMR (400 MHz, CDCl_3_): δ 2.53 (dd, *J* = 13.5, 10.2 Hz, 1H, 4-CHH), 2.79–2.94 (m, 3H, CH-4, 4-CHH, CHH-3), 3.24–3.40 (m, 1H, CHH-3), 3.58 (s, 3H, NCH_3_), 3.85 (s, 3H, OCH_3_), 6.64 (s, 1H, =CH-6), 6.86 (ddd, *J* = 8.2, 2.5, 0.8 Hz, 1H, ArH), 6,97 (t, *J* = 2.1 Hz, 1H, ArH), 7.05 (ddd, *J* = 7.8, 1.8, 0.9 Hz, 1H, ArH), 7.16–7.37 (m, 6H, ArH). ^13^C NMR (100 MHz, CDCl_3_): δ = 35.94 (CH-4), 37.33 (4-CH_2_), 42.27 (NCH_3_), 44.18 (CH_2_-3), 55.33 (O-CH_3_), 111.25, 112.68, 117.57 (ArH), 126.48 (=C-6), 126.61 (ArH), 127.98 (=C-5), 128.37, 129.61, 129.96 (ArH), 137.68, 138.57, 160.06 (Ar), 195.81 (C=S). GC-MS (EI, 70 eV): *m/z* = 323 (21) [M+], 233 (15), 232 (100), 217 (5), 189 (7), 158 (8), 115 (7), 91 (9). HRMS (ESI-TOF): *m*/*z* calcd. for C_20_H_21_NnaOS [M+Na]^+^ 346.1242; found: 346.1236.

(4*RS*)-4-Benzyl-5-(3,4-dimethoxyphenyl)-1-methyl-3,4-dihydropyridine-2(1*H*)-thione (**S17**): The crude product purified twice by column chromatography (the first column: SiO_2_, *n*-hexane: ethyl acetate, 3: 1, the second column SiO_2_, chloroform), gave yellow solid in 98% yield. Mp. 115–119 °C (*n*-hexane: ethyl acetate). ^1^H NMR (400 MHz, CDCl_3_): δ 2.54 (dd, *J* = 13.6, 9.9 Hz, 1H, 4-CHH), 2.77 (dd, *J* = 13.6, 3.4 Hz, 1H, 4-CHH), 2.82–3.03 (m, 2H, CHH-3, CH-4), 3.25–3.46 (m, 1H, CHH-3), 3.61 (s, 3H, NCH_3_), 3.92 (d, *J* = 4.6 Hz, 6H, two OCH_3_), 6.56 (s, 1H, =CH-6), 6.85–6.95 (m, 2H, C_6_H_3_), 7.03 (dd, *J* = 8.3, 2.2 Hz, 1H, C_6_H_3_), 7.12–7.48 (m, 5H, C_6_H_5_). ^13^C NMR (100 MHz, CDCl_3_): δ 36.22 (CH-4), 37.38 (4-CH_2_), 42.29 (N-CH_3_), 44.23 (CH_2_-3), 56.00 (two OCH_3_), 108.32, 108.36, 111.42, 117.91 (C_6_H_3_), 125.33 (=CH-6), 126.50 (C_6_H_5_), 128.38 (C_6_H_3_), 128.43 (C_6_H_5_), 128.98 (=C-5), 129.57 (C_6_H_5_), 138.74 (C_6_H_5_), 148.98, 149.24 (C_6_H_3_), 195.17 (C=S). GC-MS (EI, 70 eV): *m/z* = 353 (38) [M+], 264 (6), 263 (17), 262 (100), 247 (5), 246 (18), 231 (6), 206 (4), 188 (9), 115 (4), 91 (10). HRMS (ESI-TOF): *m*/*z* calcd. for C_21_H_23_NNaO_2_S [M+Na]^+^ 376.1347; found: 376.1340.

(4*RS*)-4-(Naphthalen-1-ylmethyl)-5-phenyl-3,4-dihydropyridine-2(1*H*)-thione (**S18**): The crude product purified twice by column chromatography (the first column SiO_2_, *n*-hexane: ethyl acetate, 6: 1, the second column SiO_2,_ chloroform), gave yellow solid in 68% yield. Mp. 160–162 °C (*n*-hexane: ethyl acetate). ^1^H NMR (400 MHz, CDCl_3_): δ 2.78 (dd, *J* = 16.9, 7.1 Hz, 1H, CHH-3), 2.89 (dd, *J* = 12.1, 3.0 Hz, 1H, 4-CHH), 3.22 (d, *J* = 16.9 Hz, 1H, CHH-3), 3.26–3.32 (m, 2H, 4-CHH, CH-4), 6.55 (d, *J* = 4.5, 1H, =CH-6), 7.29–7.55 (m, 9H, ArH), 6.67–7.77 (m, 1H, ArH), 7.80–7.98 (m, 2H, ArH), 9.54 (s, 1H, NH). ^13^C NMR (100 MHz, CDCl_3_): δ 34.18 (CH_2–_4), 34.48 (4-CH), 42.21 (CH_2_-3), 120.26 (=CH-6), 123.61, 125.51, 125.79, 125.92, 127.41, 127.91 (C_10_H_7_, C_6_H_5_), 128.02 (=C-5), 128.74, 128.95, 128.97, 131.69, 134.01, 134.19, 136.41 (C_10_H_7_, C_6_H_5_, two signal overlapped), 198.14 (C=S). GC-MS (EI, 70 eV); *m*/*z* = 330 (5), 329 (20), [M^+^], 189 (15), 188 (100), 187 (34), 154 (15), 142 (7), 141 (28), 128 (25), 127 (10), 115 (18). HRMS (ESI-TOF): *m*/*z* calcd. for C_22_H_20_NS [M+H]^+^ 330.1316; found: 330.1318.

(4*RS*)-4-(4-Methoxybenzyl)-5-phenyl-3,4-dihydropyridine-2(1*H*)-thione (**S19**): The crude product purified twice by column chromatography (the first column SiO_2_, *n*-hexane: ethyl acetate, 10: 1, the second column SiO_2_ chloroform), gave yellow solid in 86% yield. Mp. 149–151 °C (*n*-hexane: ethyl acetate). ^1^H NMR (400 MHz, CDCl_3_): δ 2.52 (dd, *J* = 13.9, 11.0 Hz, 1H, 4-CHH), 2.70–2.89 (m, 2H, 4-CHH, CHH-3), 2.96 (dddd, *J* = 11.0, 6.9, 3.7, 1.9 Hz, 1H, 4-CH), 3.21 (ddd, *J* = 17.0,1.9, 1.0 Hz, 1H, CHH-3), 3.79 (s, 3H, OCH_3_), 6.55 (d, *J* = 4.6 Hz, 1H, =CH-6), 6.73–6.97 (m, 2H, C_6_H_4_), 7.04–7.21 (m, 2H, C_6_H_4_), 7.27–7.35 (m, 1H, C_6_H_5_), 7.36–7.50 (m, 4H, C_6_H_5_), 9.73 (s, 1H, NH). ^13^C NMR (100 MHz, CDCl_3_): δ 35.90 (CH-4), 36.26 (4-CH_2_), 41.69 (CH_2_-3), 55.28 (OCH_3_), 113.91 (ArH), 120.10 (=CH-6), 124.97 (ArH), 127.09 (=C-5), 127.69, 128.98, 130.54, 130.55, 136.23, 158.32 (ArH, Ar), 198.01 (C=S). GC-MS (EI, 70 eV); *m*/*z* = 309 (13), [M^+^], 189 (7), 188 (43), 187 (23), 154 (12), 128 (17), 121 (100), 115 (5), 91 (5), 77 (8). HRMS (ESI-TOF): *m*/*z* calcd. for C_19_H_20_NOS [M+H]^+^ 310.1266; found: 310.1267.

4-([1,1′-Biphenyl]-4-ylmethyl)-5-phenyl-3,4-dihydropyridine-2(1*H*)-thione (**S20**): Yield 98%. The crude product purified twice by column chromatography (the first column: SiO_2_, *n*-hexane: ethyl acetate, 6: 1, the second column: SiO_2_, chloroform), gave yellow solid in 98% yield. Mp. 173–174 °C. ^1^H NMR (400 MHz, DMSO-d_6_): δ 2.43 (dd, *J* = 13.6, 11.2 Hz, 1H, 4-CHH), 2.67–2.82 (m, 2H, 4-CHH, 3-CHH), 2.91 (ddd, *J* = 17.0, 1.9, 1.0 Hz, 1H, CHH-3), 3.04–3.12 (m, 1H, CH-4), 6.75 (d, *J* = 4.6 Hz, 1H, =CH-6), 7.21–7.55 (m, 8H, ArH), 7.54–7.79 (m, 6H, ArH), 11.67 (d, *J* = 4.6 Hz, 1H, NH). ^13^C NMR (100 MHz, DMSO-d_6_): δ 34.06 (CH-4), 36.09 (4-CH_2_) 41.90 (CH_2_-3), 120.97 (=CH-6), 124.51 (Ar), 124.83 (=C-5), 126.42, 126.43, 126.95, 127.16, 128.75, 128.80, 129.89, 135.91, 137.77, 138.10, 139.84 (Ar), 196.37 (C=S). GC-MS (EI, 70 eV); *m*/*z* = 355 (15), [M^+^], 281 (8), 208, 207 (24), 189 (15), 188 (100), 187 (25), 168 (7), 154 (17), 128 (25), 127 (11). Anal. calcd for C_24_H_21_NS: C 81.09, H 5.95, N 3.94, S 9.02. Found: C 80.98, H 5.90, N 4.04.

(4*RS*)-4-Benzyl-5-(naphthalen-1-yl)-3,4-dihydropyridine-2(1*H*)-thione (**S21**): The crude product purified twice by column chromatography (the first column: SiO_2_, *n*-hexane: ethyl acetate, 6: 1, the second column SiO_2_, chloroform), gave yellow solid in 95% yield. Mp. 173–175 °C (*n*-hexane: ethyl acetate). ^1^H NMR (400 MHz, CDCl_3_): δ 2.55 (dd, *J* = 13.5, 11.4 Hz, 1H, 4-CHH), 2.76 (dd, *J* = 13.5, 3.8 Hz 1H, 4-CHH), 2.95 (dddd, *J* = 11.4, 6.6, 3.8, 3.4 Hz, 1H, CH-4), 3.08 (dd, *J* = 17.0, 6.6 Hz, 1H, CHH-3) 3.20 (dd, *J* = 17.0, 3.4 Hz, 1H, CHH-3), 6.27 (d, *J* = 4.4 Hz, 1H, =CH-6), 7.02–7.07 (m, 2H, C_6_H_5_), 7.11–7.23 (m, 3H, C_6_H_5_), 7.34 (dd, *J* = 7.0, 1.3 Hz, 1H, C_10_H_7_), 7.48 (dd, *J* = 8.3, 7.0 Hz, 1H, C_10_H_7_), 7.51–7.56 (m, 2H, C_10_H_7_), 7.86 (dt, *J* = 8.4, 1.0 Hz, 1H, C_10_H_7_), 7.89–7.95 (m, 2H, C_10_H_7_), 9.43 (s, 1H, NH). ^13^C NMR (100 MHz, CDCl_3_): δ 36.94 (4-CH_2_), 38.89 (CH-4), 41.91 (CH_2_-3), 123.00 (=CH-6), 125.08, 125.35, 126.10, 126,40, 126.48, 126.75 (C_10_H_7_), 127.56 (=C-5), 128.39, 128.49 (C_6_H_5_), 128.69 (C_10_H_7_), 129.43 (C_6_H_5_), 131.80, 133.90, 135.73 (C_10_H_7)_, 138.27(C_6_H_5_), 198.39 (C=S). GC-MS (EI, 70 eV): *m/z* = 330 (7), 329 (28), [M^+^], 240 (6), 239 (19), 238 (100), 221 (9), 205 (22), 204 (35), 179 (10), 178 (21), 152 (10), 91 (18). HRMS (ESI-TOF): *m*/*z* calcd. for C_22_H_19_NNaS [M+Na]^+^ 352.1136; found: 352.1132.

(4*RS*)-4-Benzyl-5-(thiophen-2-yl)-3,4-dihydropyridine-2(1*H*)-thione (**S22**): The crude product purified twice by column chromatography (the first column: SiO_2_, *n*-hexane: ethyl acetate), 10:1, the second column SiO_2_, chloroform), gave yellow solid in 95% yield. Mp. 130–132 °C (*n*-hexane: ethyl acetate). ^1^H NMR (400 MHz, CDCl_3_) δ 2.60 (dd, *J* = 14.9, 12.0 Hz, 1H, 4-CHH), 2.78 (dd, *J* = 17.1, 6.9 Hz, 1H, CHH-3), 2.85–2.97 (m, 2H, 4-CHH, CH-4), 3.19 (d, *J* = 17.1 Hz, 1H, CHH-3), 6.58 (d, *J* = 4.5 Hz, 1H, =CH-6), 7.03 (dd, *J* = 5.1, 3.6 Hz, 1H, C_4_H_3_S), 7.08 (dd, *J* = 3.7, 1.2 Hz, 1H, C_4_H_3_S), 7.20–7.28 (m, 4H, C_4_H_3_S, C_6_H_5_), 7.29–7.35 (m, 2H, C_6_H_5_), 9.78 (s, 1H, NH). ^13^C NMR (101 MHz, CDCl_3_) δ 37.28 (CH-4), 37.32 (4-CH_2_), 41.53 (CH_2_-3), 118.82 (=CH-6), 122.05 (=C-5), 122.96, 124.20 (C_4_H_3_S), 126.62 (C_6_H_5_), 127.91 (C_4_H_3_S), 128.55 (2C, C_6_H_5_), 129.58 (2C, (C_6_H_5_), 138.43 ((C_6_H_5_), 140.61 (C_4_H_3_S), 197.42 (C=S). GC-MS (EI, 70 eV): *m/z* = 285 (48), [M^+^], 195 (13), 194 (100), 193 (14), 161 (23), 134 (30), 91 (21). HRMS (ESI-TOF): *m*/*z* calcd. for C_16_H_16_NS_2_ [M+H]^+^ 286.0724; found: 286.0724.

### 4.4. Cell Culture

Human breast adenocarcinoma cells (MCF7 ECACC 86012803), malignant melanoma cells (A375 ECACC 88113005), colon adenocarcinoma (HT-29 ECACC 91072201), ovary adenocarcinoma (SK-OV-3 ECACC 91091004) and prostate adenocarcinoma (PC-3 ECACC 90112714) were purchased from European Collection of Authenticated Cell Cultures (ECACC). Human Epidermal Melanocytes HEM (neonatal, 104K-05n) were purchased from Cells Applications (San Diego, CA, USA). All cells were cultured in a humidified incubator (5% CO_2_, 37 °C) in recommended culture medium supplemented with heat-inactivated fetal bovine serum (FBS, EURx, Gdansk, Poland), L-glutamine (2 mM, Sigma-Aldrich Merck Group, St. Louis, MO, USA) and penicillin-streptomycin (Sigma-Aldrich Merck Group, St. Louis, MO, USA). Cell lines were routinely tested for the presence of mycoplasma.

### 4.5. Antiproliferative Activity

The antiproliferative activity of **S1** and **S1** analogues (**S2**-**S22**), with combretastatin (CA-4, Sigma-Aldrich Merck Group, St. Louis, MO, USA) as reference, were evaluated using the Cell Proliferation Reagent WST-1 assay (Sigma-Aldrich Merck Group, St. Louis, MO, USA). It should be noted that this kit is commonly used for proliferation measurement, but it does not directly measure DNA replication, and may be influenced by cell metabolic activity. WST-1 assay is based on the reduction of the tetrazolium salt to formazan which amount is directly correlated to the number of metabolically active cells. In the present study, MCF7, A375, HT-29, SK-OV-3 and PC-3 cells were seeded in 96-well plate (at plating densities depending on the doubling time of individual cell lines) and then cultured in appropriate medium in standard conditions. After 24 h the culture medium was removed and replaced with fresh medium containing compounds at final concentrations equal: 1, 10, 50, 100 µM for 48 h. All the tested compounds were dissolved in DMSO purchased from Sigma-Aldrich Merck Group, St. Louis, MO, USA (final concentrations of DMSO did not exceed 0.2%; initially impact of 0.2% DMSO on cell viability was negatively verified). The cells without tested compounds (but in medium with DMSO) were used as control and tested compounds in medium without cells as blank. After 48 h, WST-1 reagent was added, incubated with the cells for 30 min and absorbance was measured at 450 nm (with 620 nm background correction), using a spectrophotometric microplate reader (Infinite 200 Pro, Tecan, Männedorf, Switzerland). The cell viability was calculated using the following formula: [(Atest − Ablank)/(Acontrol − Ablank)] × 100%. The readings were acquired from at least three independent experiments (each conducted in triplicate). The IC_50_ values (the inhibitory concentration causing 50% growth inhibition) were evaluated using an online calculator (AAT Bioquest, Inc., Quest Graph™ IC50 Calculator (v.1). Retrieved from: https://www.aatbio.com/tools/ic50-calculator-v1 (accessed on: 10 September 2020).

### 4.6. Selectivity-Index

The malignant melanoma (A375) and non-tumor melanocytes (HEM) cells were used to estimate the anticancer selectivity of the three the most active compounds from the earlier study (**S1**, **S19** and **S22**; CA-4 was used as reference). The WST-assay and IC_50_ calculation were performed, as described above. Then the selectivity index (SI) was calculated according to the following formula: SI = IC_50_ normal cells/IC_50_ cancer cells.

### 4.7. Apoptosis Detection 

A375 and HEM cells were seeded in 24-well plates (1 × 10^4^ and 2 × 10^4^ cells/well, respectively). After 24 h, the culture medium was removed and the cells were treated with **S1**, **S19**, **S22** and CA-4 (as reference) at final concentrations of 10 µM and incubated for another 48 h. Untreated cells growing in medium with DMSO (0.2%) was used as control. After treatment the cells were washed with PBS (both the cell medium and PBS were collected) and harvested by trypsinization. After centrifugation, the supernatant was discarded and cells pellet was resuspended in appropriate medium. Next, equal volume of the Muse Annexin V & Dead Cell Reagent (Luminex Corporation, Austin, TX, USA) was added, mixed thoroughly by pipetting up and down and stained for 20 min (at RT in the dark). Samples were analyzed by Muse Cell Analyzer (Luminex Corporation, Austin, TX, USA). The assay is based on the detection of phosphatidylserine (PS) on the surface of apoptotic cells by binding with Annexin V-PE. A dead cell marker (7-AAD) is also used as an indicator of cell membrane structural integrity. Simultaneous staining with these two dyes allow to distinguish four populations of cells: viable (Annexin V-PE− and 7-AAD−), early apoptotic (Annexin V-PE+ and 7-AAD−), late apoptotic/already dead cells (Annexin V-PE+ and 7-AAD+), mostly nuclear debris (Annexin V-PE− and 7-AAD+). According to the manufacturer’s protocol (on the base of cell size index) debris were excluded from further analysis. The readings were acquired from at least three independent experiments. Additionally, optical microscopy imaging of A375 and HEM cells was performed just before apoptosis analysis of harvested cells using Smart Fluorescent Cell Analyzer Microscope JuLi (Seoul, Korea). 

### 4.8. Cell Cycle Analysis

For cell cycle analysis, A375 cells were seeded in 6-well plates (1 × 10^5^ cells/well) and cultured for 24 h. Then the medium was removed and replaced with fresh medium containing **S1**, **S19**, **S22** and CA-4 at final concentrations of 10 µM and incubated for additional 6 and 24 h. Afterwards, cells were washed with PBS and harvested by trypsinization. Then, cell pellet was washed with PBS and fixed with 70% cold ethanol at −20 °C for at least 3 h. Before the analysis ethanol-fixed cells were centrifuged, washed with PBS and stained with Muse Cell Cycle Reagent (Luminex Corporation, Austin, TX, USA), containing propidium ioide (PI) and RNAse, for 30 min at RT in the dark. The assay utilizes PI-based staining of DNA content to discriminate and measure the percentage of cells in each cell cycle phase (G0/G1, S, and G2/M) using a Muse Cell Analyzer (Luminex Corporation, Austin, TX, USA). The readings were acquired from three independent experiments.

### 4.9. Confocal Microscopy Imaging

To visualize the microtubule and DNA, A375 cells were seeded on cover slips at density of 1 × 10^5^ cells/well and cultured as above for 24 h. Afterwards, 10 µM of compounds: **S1**, **S2** and **S22** were added, and incubated for additional 6 h. Next, the cells were fixed in 4% buffered formalin for 5 min at 37 °C, washed twice with PBS and permeabilized with 0.01% Triton X-100 (Sigma-Aldrich Merck Group, St. Louis, MO, USA) in PBS for 10 min at RT. Nonspecific antibody binding was blocked (0.01% Triton X-100, 2% BSA, 1.5% FBS in PBS) for 10 min (RT) and subsequently incubated with mouse anti-α-tubulin monoclonal antibody (Sigma-Aldrich Merck Group, St. Louis, MO, USA), diluted 1:2000 for overnight (4 °C). The cover slips were then washed tree times with 0.05% Triton X-100 in PBS and the cells were incubated with secondary antibodies anti-mouse IgG-FITC (Sigma-Aldrich Merck Group, St. Louis, MO, USA) diluted 1:64 for 1 h at RT, washed and counterstained with DAPI (Sigma-Aldrich Merck Group, St. Louis, MO, USA). Finally, cells were washed with PBS and mounted with mounting medium (Dako-Agilent, CA, USA) on glass slides. The slides were examined with a FV1000 confocal microscope (Olympus, Hamburg, Germany) in two separated channels: for DAPI (405 nm laser), FITC (488 nm laser). To calculate the proportion of different mitotic spindle phenotypes cells in mitosis from ten random fields (×20 objective magnification) of the slide were counted (from two independent experiments). 

### 4.10. Cell Free Tubulin Polymerization Assay

Tubulin polymerization was monitored using a >99% pure tubulin and fluorescence-based tubulin polymerization kit (Cytoskeleton, Denver, CO, USA), where tubulin polymerization is followed by fluorescence enhancement due to the incorporation of a fluorescence reporter into microtubules as polymerization proceeds. Compounds **S1**, **S19** and **S22** (at final concentrations: 100, 50, 10 and 1 µM) were evaluated for their effect on tubulin polymerization (in cell free conditions). Paclitaxel (Cytoskeleton, Denver, CO, USA) and CA-4 (10 µM) were used as references and DMSO (0.2%) as vehicle control. According to the manufacturer’s protocol after incubation of tested compounds at 37 °C for 1 min, the icy tubulin reaction mixture (2 mg/mL tubulin, 1.0 mM GTP, 15% glycerol in buffer containing 80 mM PIPES, 2.0 mM MgCl2, 0.5 mM EGTA, pH 6.9 and 10 µM fluorescent reporter) was added. The samples were mixed and tubulin assembly was monitored (excitation: 360 nm, emission: 450 nm) at 1 min intervals for 1 h at 37 °C using a spectrophotometric microplate reader (Infinite 200 Pro, Tecan, Männedorf, Switzerland). The inhibition of tubulin polymerization was expressed as IC_50_ values and quantified according to Cytoskeleton, Inc. instruction. Firstly, maximal slope (V max) of the growth phase expressed as RFU/min was calculated using a software installed on the aforementioned plate reader. Next, V max of each polymerization curve was normalized to V max of appropriate control’s curve and IC_50_ values were evaluated using an online calculator (AAT Bioquest, Inc. Quest Graph™ IC50 Calculator. Retrieved from https://www.aatbio.com/tools/ic50-calculator (accessed on: 16 February 2021).

### 4.11. Cell-Based Tubulin Polymerization Assay

Western blot based tubulin polymerization assay was modified by previously described method [57,58]. A375 cells were seeded in 6-well plates (5 × 10^4^ cells/well), grown to subconfluency and next additional 6 h with 10 µM of compounds **S1**, **S19**, **S22**, CA-4 and PTX (as references), and DMSO (0.2%) as control. After treatment, cells were washed twice with warm PBS, lysed shaking for 5 min with hypotonic buffer (20 mM Tris–HCl pH 6.8, 1 mM MgCl_2_, 2 mM, EGTA, 0.5% NP-40; Sigma-Aldrich Merck Group, St. Louis, MO, USA) containing 1X protease inhibitor cocktail (Thermo Fisher Scientific, Waltham, MA, USA), then scraped and transferred to tubes. The samples were centrifuged at 13,200 rpm for 10 min at RT, and supernatants containing soluble (cytosolic) tubulin were separated from the pellets containing polymerized (cytoskeletal) tubulin. The pellets were resuspended in equal volumes of hypotonic buffer. Total protein concentration was then determined in each fraction by BCA method (Thermo Fisher Scientific, Waltham, MA, USA). The equal amount of protein (6 µg per well) from supernatant and pellet fractions were resuspended in 2× Laemmli buffer (Bio-Rad, Hercules, CA, USA), heated for 5 min at 95 °C and analyzed by SDS-PAGE using 4–20% mini-PROTEAN electrophoresis system (Bio-Rad, Hercules, CA, USA), then transferred to PVD membrane (Bio-Rad, Hercules, CA, USA). Immunoblotting was performed using a primary mouse anti-α-tubulin monoclonal antibody (Sigma-Aldrich Merck Group, USA) diluted 1:1000 for overnight (4 °C) and secondary mouse IgGκ BP conjugated to horseradish peroxidase (HRP) (Santa Cruz Biotechnology, Dallas, TX, USA) diluted 1:2000. The obtained products were detected using the Western Bright Sirius Chemiluminescent Detection Kit (Advansta, San Jose, CA, USA) and bands were subsequently visualized in UVP camera (UVP BioImaging system EpiChemi 3, Houston TX, USA). Equal loading in the lanes was evaluated by stripping the blots according to manufacturer’s protocol (Restore Western Blot Stripping Buffer, Thermo Fisher Scientific, Waltham, MA, USA) and reprobing them with mouse β-actin monoclonal antibody 1:1000 (Santa Cruz Biotechnology, Dallas, TX, USA) and then secondary HRP-conjugated antibody (as above). Percentage of α-tubulin in the pellet fractions was calculated as a densitometric value of the pellet band divided by the total densitometric value of the pellet and supernatant bands using ImageJ Software 1.53c (Maryland, VA, USA). Each densitometric value of tubulin was before normalized to the densitometric value of β-actin The readings were acquired from four independent experiments.

### 4.12. Colchicine Binding-Site Assay

Colchicine binding-site assay was performed as previously described [44] by using >99% pure tubulin protein (Cytoskeleton, Denver, CO, USA) reconstituted in buffer containing 80 mM PIPES pH 6.9, 2 mM MgCl_2_, 0.5 mM EGTA supplemented with 1 mM GTP. The compounds **S1**, **S19** and **S22** at 50 and 10 µM were incubated with 3 µM tubulin in the presence and absence of 3 µM colchicine (Sigma-Aldrich Merck Group, St. Louis, MO, USA) in 30 mM Tris buffer for 60 min at 37 °C. CA-4 (10 µM) was used as a positive control whereas vinblastine (VBL, Sigma-Aldrich Merck Group, St. Louis, MO, USA) binding at distinct site on tubulin (10 µM) was used as negative control. This method is based on the fact that the intrinsic fluorescence of colchicine increases upon binding to tubulin. Hence, tubulin binding site inhibitors that compete with colchicine may decrease fluorescence of colchicine-tubulin complex. After incubation fluorescence was determined (excitation 350 nm, emission 435 nm) by using a spectrophotometric microplate reader (Infinite 200 Pro, Tecan, Männedorf, Switzerland). The raw fluorescence values were normalized first by subtracting the fluorescence of the blank (tubulin with tested compounds without colchicine) and setting the fluorescence of 3 μM tubulin with 3 μM colchicine to 100% (control with 0.2% DMSO). 

### 4.13. Molecular Docking

Computational docking was carried out using the genetic algorithm-based ligand docking program Chimera and AutoDock Vina version 1.1.2 (San Diego, CA, USA). The X-ray crystal structure of αβ-tubulin complexed colchicine (PDB code: 4o2b) was used in this study. The stathmin-like domain and the C and D subunits were removed from the model. Binding modes for each colchicine analogue were investigated to understand the steric, electrostatic, and hydropathic features of the colchicine binding site. The top 10 conformations with different scores (between −5 to −9) were acquired, and the best ranking pose was visualized.

### 4.14. Statistical Analysis

Results are presented as mean ± standard deviation (SD). Statistical analysis was carried out using Statistica 13.3 (StatSoft Inc., Tulsa, OK, USA) and ANOVA test followed by a post hoc Tukey’s multiple comparison. A *p*-value level of < 0.05 was considered statistically significant.

## Figures and Tables

**Table 1:** The IC_50_ values (μM) determined using WST-1 assay after 48 h of treatment. Data are expressed as the mean ± SD from at least three independent experiments.

Compounds	MCF7	A375	HT-29	SK-OV3	PC-3	LogP ^1^
**S1**	18.02 ± 9.01	4.33 ± 1.00	39.22 ± 17.59	6.75 ± 2.88	50.66 ± 0.61	3.86
**S2**	70.03 ± 7.30	12.55 ± 2.16	89.46 ± 9.78	18.06 ± 4.31	>100	4.10
**S3**	84.87 ± 0.01	53.89 ± 16.80	>100	68.94 ± 14.18	>100	4.91
**S4**	>100	>100	>100	>100	>100	5.88
**S5**	>100	>100	>100	>100	>100	2.49
**S6**	99.08 ± 0.92	97.51 ± 0.86	>100	74.71 ± 1.58	≥100	4.09
**S7**	74.22 ± 15.44	82.82 ± 9.99	>100	54.95 ± 20.42	≥100	4.45
**S8**	34.18 ± 2.86	22.44 ± 1.79	46.61 ± 4.88	34.68 ± 15.00	48.57 ± 1.22	4.00
**S9**	45.81 ± 3.4	24.38 ± 4.79	51.57 ± 2.95	51.01 ± 1.21	48.41 ± 3.26	4.13
**S10**	34.25 ± 8.07	17.41 ± 2.00	40.38 ± 12.25	31.62 ± 14.33	40.28 ± 8.47	4.37
**S11**	40.06 ± 16.45	8.33 ± 2.02	42.44 ± 6.37	14.39 ± 2.5	50.50 ± 6.82	3.60
**S12**	57.77 ± 9.94	53.94 ± 7.29	53.31 ± 4.48	61.66 ± 11.94	37.46 ± 11.53	3.35
**S13**	>100	41.89 ± 9.18	>100	42.93 ± 3.25	>100	4.24
**S14**	>100	>100	>100	>100	>100	4.38
**S15**	>100	>100	>100	>100	>100	4.62
**S16**	>100	18.83 ± 3.93	>100	38.71 ± 12.79	>100	3.85
**S17**	>100	>100	>100	>100	53.31 ± 28.82	3.60
**S18**	24.45 ± 5.3	10.18 ± 1.52	27.66 ± 2.88	30.32 ± 8.81	23.07 ± 8.93	4.10
**S19**	20.15 ± 2.14	3.70 ± 1.26	21.33 ± 3.45	13.40 ± 3.26	31.47 ± 13.98	3.60
**S20**	35.90 ± 9.69	9.91 ± 3.29	55.46 ± 5.21	39.57 ± 7.73	57.60 ± 1.08	5.54
**S21**	40.48 ± 14.95	10.89 ± 2.69	>100	15.86 ± 5.94	72.66 ± 8.73	4.86
**S22**	44.86 ± 17.1	1.71 ± 0.58	16.54 ± 6.86	1.67 ± 1.47	20.91 ± 2.13	3.15
CA-4	0.008 ± 0.003	0.002 ± 0.001	>10	0.005 ± 0.001	0.004 ± 0.004	3.38

^1^ Caclulated using the Ghose–Crippen approach [18]

**Table 2:** Selectivity-index (SI) for selected compounds.

Compounds	A375	HEM	SI ^1^
**S1**	4.33 ± 1.00	28.80 ± 7.77	6.65
**S19**	3.70 ± 1.26	33.95 ± 0.33	9.18
**S22**	1.71 ± 0.58	36.11 ± 4.03	21.09
CA-4	0.002 ± 0.001	0.001 ± 0.0004	0.57

^1^ Selectivity index: IC_50_ normal cells/IC_50_ cancer cell.

**Table 3:** The IC_50_ values (μM) of inhibition of tubulin polymerization. Data are expressed as the mean ± SD from at least three independent experiments.

Compounds	IC_50_^1^
**S1**	10.77 ± 3.83
**S19**	17.38 ± 10.00
**S22**	26.82 ± 15.21
CA-4 [44]	1.08 ± 0.33

^1^ Concentration of compound required to inhibit tubulin assembly by 50%, calculated on the base of maximal slope (V max) of the growth phase polymerization curve and normalized to control.

## Data Availability

Not applicable.

## Supplementary Materials

The following are available online at https://www.mdpi.com/1422-0067/22/5/2462/s1, Figure S1. Representative scatter diagrams of A375 cells exposed to medium with 0.2% DMSO (A), CA-4 (B), **S1** (C), **S19** (D), **S22** (E) at 10 µM for 48 h. Figure S2. Representative scatter diagrams of HEM cells exposed to medium with 0.2% DMSO (A), CA-4 (B), **S1** (C), **S19** (D), **S22** (E) at 10 µM for 48 h; Figure S3: Optical microscopy images of A375 cells, controls (A) and after 48 h incubation with 10 µM of compounds: CA-4 (B), **S1**(C), **S19** (D) and **S22** (E); Figure S4: Optical microscopy images of HEM cells, controls (A) and after 48 h incubation with 10 µM of compounds: CA-4 (B), **S1**(C), **S19** (D) and **S22** (E); Figure S5. Representative individual experiment of cell cycle phase distribution of A375 cells exposed to medium with 0.2% DMSO (A), CA-4 (B), S1 (C), S19 (D), S22 (E) for 6 h and DMSO (F), CA-4 (G), S1 (H), S19 (I), S22 (J) for 24 h. Figure S6: Original western blot images for Figure 8 (wells shown as white). A, C-α-tubulin, B,D- β-actin used as a loading control in stripped and reprobed membrane; Figure S7: Binding model of compound **S1** at the colchicine binding-site of tubulin. The hydrogen bond is shown as blue line; Figure S8: Binding model of compound **S19** at the colchicine binding-site of tubulin. The hydrogen bonds are shown as blue line; Figure S9: First possible binding model of compound **S22** at the colchicine binding-site of tubulin. The hydrogen bond is shown as blue line; Figure S10: Second possible binding model of compound **S22** at the colchicine binding-site of tubulin. The hydrogen bond is shown as blue line.

## Appendix A

### Appendix A.1. Synthesis of Precursors of Compounds **S1**-**S22**

#### Synthesis and Spectroscopic Data of Compounds **1**-**8**

Synthesis of 5-Ar-2-methoxypyridines (**1**-**7**) were performed from 5-bromo-2-methoxypyridine according to procedure described earlier, [59] with some modifications:

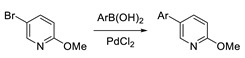

To the mixture of DMF (60 mL) and water (60 mL) in a 250 mL flask 5-bromo-2-methoxypyridine (3 g, 16.0 mmol), arylboronic acid (18.4 mmol), (K_3_PO_4_^.^5H_2_O (3.19 g; 12 mmol) and PdCl_2_ (0.057 g; 0.32 mmol). The resulting solution was stirred for 24 h at room temperature in open flask. After this time brine (100 mL) was added and the aqueous layer was extracted with diethyl ether (4 × 100 mL) and the organic layer were washed with brine and dried with MgSO_4_. The mixture was filtered, and the solvents were evaporated under reduced pressure. The crude product was purified by column chromatography on SiO_2_.

5-(4-Fluorophenyl)-2-methoxypyridine (**2**): The crude product purified by column chromatography (SiO_2_, *n*-hexane: ethyl acetate 11:1), gave white solid in 86% yield. Mp. 76–77 °C (petroleum ether: ethyl acetate). ^1^H and ^13^C NMR spectra of **2** matched those previously reported [60].

5-(3,4-Difluorophenyl)-2-methoxypyridine (**3**): The crude product purified by column chromatography (SiO_2_, *n*-hexane: ethyl acetate 10:1), gave white solid in 96% yield. Mp. 86–88 °C (petroleum ether: ethyl acetate). ^1^H NMR (400 MHz, CDCl_3_) δ 3.98 (s, 3H, OCH_3_), 6.82 (dd, *J* = 8.6 Hz, 1H, CH-3), 7.15–7.37 (m, 3H, C_6_H_3_F_2_), 7.71 (dd, *J* = 8.6, 2.6 Hz, 1H, CH-4), 8.32 (d, *J* = 2.6 Hz, 1H, CH-6). ^13^C NMR (101 MHz, CDCl_3_) δ 53.65 (O-CH_3_), 111.05 (CH-3), 115.60 (d, *J* = 17.8 Hz, CH-2′), 117.83 (d, *J* = 17.6 Hz, CH-5′), 122.62 (dd, *J* = 6.2, 3.5 Hz, CH-6′), 128.19 (C-5), 135.08 (dd, *J* = 6.2, 3.9 Hz, C-1′), 137.24 (CH-4), 144.91 (CH-6), 149.94 (dd, *J* = 248.6, 12.3 Hz, C-3′), 150.65 (dd, *J* = 248.2, 13.3 Hz, C-4′), 163.92 (C-2). GC-MS (EI, 70 eV): *m*/*z* = 221 (100), [M+], 220 (97), 192 (45), 190 (46), 164 (15), 151 (34), 138 (9). HRMS (ESI-TOF): *m*/*z* calcd. for C_12_H_10_F_2_NO [M+H]^+^ 222.0730; found: 222.0728.

5-(4-Chlorophenyl)-2-methoxypyridine [61] (**4**): The crude product purified by column chromatography (SiO_2_, *n*-hexane: ethyl acetate 11:1), gave white semisolid in 90% yield. ^1^H NMR (400.1 MHz, CDCl_3_): δ 3.98 (3 H, s, OCH_3_), 6.81 (dd, *J* = 8.6, 0.7 Hz,1H CH-3), 7.3 -7.49 (m, 4H, C_6_H_5_), 7.74 (dd, *J* = 8.6, 2.5 Hz, 1H, CH-4), 8.35 (dd, *J* = 2.5, 0.8 Hz, 1H, CH-6). ^13^C NMR (100.6 MHz, CDCl_3_): δ 53.59 (OCH_3_), 110.95 (CH-3), 127.89 (C_6_H_5_), 128.91 (C-5), 129.13 (2× C_6_H_5_), 133.44, 136.38 (C_6_H_5_), 137.23 (CH-4), 144.89 (CH-6), 163.78 (C-2). GC-MS (EI, 70 eV): *m*/*z* = 221 (33), 220 (44), 218 (100), 191 (18), 190 (55), 189 (29), 188 (18), 154 (30), 140 (10), 127 (16), 114 (16), 75 (10), 63 (14).

2-Methoxy-5-(3-methoxyphenyl)pyridine [62] (**5**): The crude product purified by column chromatography (SiO_2_, *n*-hexane: ethyl acetate 15:1), gave colorless oil in 91% yield. ^1^H NMR (400.1 MHz, CDCl_3_): δ 3.85 (s, 3H, OCH_3_), 3.98 (s, 3H, OMe), 6.80 (dd, *J* = 8.6, 0.8 Hz,1H, CH-3), 6.89 (ddd, *J* = 8.2, 2.6, 1.0 Hz, 1H, C_6_H_4_), 7.05 (dd, *J* = 2.6, 1.7 Hz, 1H, C_6_H_4_), 7.10 (dt, *J* = 7.7, 1.3 Hz, 1H, C_6_H_4_), 7.35 (dd, *J* = 8.2, 7.7 Hz, 1H, C_6_H_4_), 7.77 (dd, *J* = 8.6, 2.6 Hz, 1H, CH-4), 8.39 (dd, *J* = 2.6, 0.8 1H, CH-6). ^13^C NMR (100.6 MHz, CDCl_3_): δ = 55.54 (OCH_3_), 55.28 (OCH_3_), 110.78 (CH-3), 112.53, 112.56, 119.15 (C_6_H_4_), 129.94 (=C-5), 130.00 (C_6_H_4_), 137.47 (CH-4), 139.38 C_6_H_4_), 145.02 (CH-6), 160.07 (C_6_H_4_), 163.69 (C-2). GC-MS (EI, 70 eV): *m*/*z* = 216 (17), 215(100) [M^+^], 186 (42), 185 (27), 184 (12), 171 (12), 154 (11), 142 (11), 115 (17), 102 (15), 93 (9).

5-(3,4-Dimethoxyphenyl)-2-methoxypyridine [63] (**6**): The crude product purified by column chromatography (SiO_2_, *n*-hexane: ethyl acetate 3:1), gave colorless solid in 93% yield. Mp. 85–86 °C (petroleum ether:ethyl acetate). ^1^H NMR (400.1 MHz, CDCl_3_): δ 3.92 (s, 3H, OCH_3_), 3.94 (s, 3H OCH_3_), 3.98 (s, 3 H, OCH_3_), 6.80 (dd, *J* = 8.5, 0.7 Hz,1H, CH-3), 6.95 (d, *J* = 8.2, 1H, C_6_H_3_), 7.02 (d, *J* = 2.1 Hz, 1H, C_6_H_3_), 7.07 (dd, *J* = 8.2, 2.1 Hz, 1H, C_6_H_3_), 7.75 (dd, *J* = 8.6, 2.6 Hz, 1H, CH-4), 8.35 (dd, *J* = 2.5, 0.6 Hz, 1H, CH-6). ^13^C NMR (100.6 MHz, CDCl_3_): δ 55.52, 55.96, 56.01 (three OCH_3_), 110.03 (C_6_H_3_), 110.72 (CH-3), 111.68, 119.01 (C_6_H_3_), 130.03 (C-5), 130.89 (C_6_H_3_), 137.28 (CH-4), 144.64 (CH-6), 148.67, 149.35 (C_6_H_3_), 163.78 (C-2). GC-MS (EI, 70 eV); *m*/*z* = 246 (16), 245 (100), [M^+^], 230 (26), 202 (19), 177 (17), 170 (8), 159 (8).

2-Methoxy-5-(naphthalen-1-yl)pyridine [64] (**7**): The crude product purified by column chromatography (SiO_2_, *n*-hexane: ethyl acetate 10:1), gave white semisolid in 89% yield. ^1^H NMR (400.1 MHz, CDCl_3_): δ 4.02 (s, 3 H, OCH_3_), 6.87 (dd, *J* = 8.4, 0.8 Hz, 1 H, CH-3), 7.34–7.57 (m. 4 H, C_10_H_7_), 7.70 (dd, *J* = 8.4, 2.4 Hz, 1H, CH-4), 7.79–7.99 (m, 3H, C_10_H_7_), 8.29 (dd, *J* = 2.4, 0.8 Hz, 1H, CH-6). ^13^C NMR (100.6 MHz, CDCl_3_): δ 53.55 (OCH_3_), 110.35 (CH-3), 125.49 (=C-5), 125.49, 125.95, 126.31, 127.22, 128.05, 128.42, 129.45, 131.81, 133.84, 136.40 (C_10_H_7_), 140.33 (CH-4), 147.25 (CH-6), 163.52 (C-2). GC-MS (EI, 70 eV); *m*/*z* = 235 (100) [M^+^], 234 (74), 206 (33), 204 (35), 191 (9), 165 (24), 102 (13), 88 (8).

2-Methoxy-5-phenylpyridine [59] (**1**) was prepared on multigram scale as follows:

To the mixture of DMF (300 mL) and water (300 mL) in a 1 l flask 5-bromo-2-methoxypyridine (20 g, 0.106 mol), arylboronic acid (14.3 g, 0.117 mol), (K_3_PO_4_^.^5H_2_O (22 g; 0.0827 mol) and PdCl_2_ (0.374 g, 2.1 mmol). The resulting solution was stirred for 22 h at room temperature in open flask. After this time brine (300 mL) was added and the aqueous layer was extracted with diethyl ether (4 × 300 mL) and the organic layer were washed with brine (100 mL) and dried with MgSO_4_. The mixture was filtered, and the solvents were evaporated under reduced pressure. The crude product was purified by vacuum distillation (bp 68–70 °C, 0.01 mbar) afforded **1** in 95% yield (18.57 g) as colorless oil. ^1^H and ^13^C NMR spectra matched those previously reported [65].

2-Methoxy-5-(thiophen-2-yl)pyridine [66] (**8**) was obtained as follows:

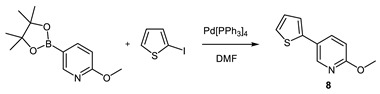

To a 100-mL Schlenk flask, equipped with a magnetic stir bar and a condenser crowned with argon balloon, charged with 40 mL of degassed DMF, 2-iodothiophene (Apollo Scientific, 1 g, 4.76 mmol), 2-methoxy-5-(4,4,5,5-tetramethyl-1,3,2-dioxaborolan-2-yl)pyridin [67] (1.679 g, 7.14 mmol, 1.5 eqiv.) and 0.275 g tetrakis(triphenylphosphino)palladium(0) (Sigma-Aldrich Merck Group, St. Louis, MO, USA) was added, and the mixture was stirred at rt for 60 min. Next 21.5 mL of degassed 1.0 M aqueous Na_2_CO_3_ solution was added, and the reaction mixture was heated under argon at 80 °C for 3 h. After this time the reaction mixture was cooled to rt. and solvent was removed under reduced pressure. The residue was extracted by ethyl acetate, and the organic layer was washed with brine, and dried over MgSO_4._ The crude product was purified by column chromatography on silica gel using a mixture *n*-hexane and ethyl acetate (9:1 *v*/*v*) as the eluent, yielding **8** as yellow oil 0.827g (90%).

^1^H NMR (400 MHz, CDCl_3_) δ 3.96 (s, 3H, OCH_3_), 6.76 (dd, *J* = 8.6, 0.8 Hz, 1H, CH-3), 7.07 (dd, *J* = 5.1, 3.5 Hz, 1H, C_4_H_3_S), 7.21 (dd, *J* = 3.5, 1.2 Hz, 1H, C_4_H_3_S), 7.27 (dd, *J* = 5.1, 1.2 Hz, 1H, C_4_H_3_S), 7.76 (dd, *J* = 8.6, 2.6 Hz, 1H, CH-4), 8.42 (d, *J* = 2.6 Hz, 1H, CH-6). ^13^C NMR (101 MHz, CDCl_3_) δ 53.59 (t), 110.94 (d), 122.93 (d), 124.10 (s), 124.66 (d), 128.06 (d), 136.54 (d), 140.77 (s), 143.96 (d), 163.56 (s). GC-MS (EI, 70 eV); *m*/*z* = 191 (100) [M^+^], 190 (64), 162 (57), 161 (25), 160 (25), 148 (12), 121 (45), 77 (12).

Synthesis of compounds **1**-**8**(**NH**):

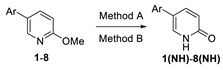

**Method A:** A mixture of 2-methoxy(5-aryl)pyridine (0.016 mol) and pyridine hydrochloride (18.4 g, 0.16 mol) was heated in a 100-mL flask at 160 °C (oil bath) on continuous stirring for 20 min. After cooling to rt brine (40 mL) was added followed with of water (20 mL). White solid was filtered off, dissolved in ethyl acetate and passed through a pad of SiO_2_ and washed with ethyl acetate till complete product was obtained (TLC-control) [**1**-**2**(**NH**)] or chromatographed on SiO_2_ (ethyl acetate) [**3**-**4**(**NH**)], [**7**-**8**(**NH**)].

5-Phenylpyridin-2(1*H*)-one [60] [**1**(**NH**)]: Yield 84%. Mp.177–178 °C. ^1^H and ^13^C NMR spectra matched those previously reported [60].

5-(4-Fluorophenyl)pyridin-2(1H)-one [60] [**2**(**NH**)]: Yield 94%. Mp. 178–180 °C (petroleum ether:ethyl acetate). ^1^H and ^13^C NMR spectra matched those previously reported [60].

5-(3,4-Difluorophenyl)pyridin-2(1*H*)-one [**3**(**NH**)]: Yield 90%. Mp. 210–213 °C (petroleum ether:ethyl acetate). ^1^H NMR (400 MHz, DMSO-*d*_6_) δ 6.57 (d, *J* = 9.8 Hz, 1H, CH-3), 6.97–7.42 (m, 3H, C_6_H_3_F_2_), 7.53 (d, *J* = 2.9 Hz, 1H, CH-6), 7.66 (dd, *J* = 9.8, 2.9 Hz, CH-4), 12.05 (br s, 1H, NH). ^13^C NMR (101 MHz, DMSO-*d*_6_) δ 114.49 (d, *J* = 17.9 Hz, CH-2′), 117.49 (C-5), 117.73 (d, *J* = 17.4 Hz, CH-5′), 120.75 (CH-3), 121.66 (dd, *J* = 6.2, 3.3 Hz, CH-6′), 132.43 (CH-6), 133.85 (dd, *J* = 6.2, 3.7, Hz, C-1′), 139.98 (CH-4), 149.20 (dd, *J* = 248.9, 12.2 Hz, C-3′), 150.12 (dd, *J* = 248.9, 12.2 Hz, C-4′), 162.92 (C=O). GC-MS (EI, 70 eV); *m*/*z* = 207 (100), [M^+^], 179 (65), 151 (72), 125 (8). HRMS (ESI-TOF): *m*/*z* calcd. for C_11_H_8_F_2_NO [M+H]^+^ 208.0574; found: 208.0565.

5-(4-Chlorophenyl)pyridin-2(1*H*)-one [68] [**4**(**NH**)]: Yield 96%. Mp. 185–186 °C (ethyl acetate: petroleum ether). ^1^H NMR (400.1 MHz, CDCl_3_): δ 6.70 (1 H, d, *J* = 9.4 Hz, CH-3), 7.32–7.43 (m, 4 H, C_6_H_4_), 7.62 (d, *J* = 2.7 Hz, 1H, CH-6), 7.75 (dd, *J* = 9.4, 2.6 Hz, 1 H, CH-4), 13.37 (s, 1 H, br, NH). ^13^C NMR (100.6 MHz, CDCl_3_): δ 120.18 (C=5), 120.45 (CH-3), 127.04, 129.31 (C_6_H_4_), 132.05 (CH-6), 133.50, 134.79 (C_6_H_4_), 141.15 (CH-4), 164.80 (C=O). GC/MS decomposition.

5-(Naphthalen-1-yl)pyridin-2(1*H*)-one [**7**(**NH**)]: Yield 77%. Mp. 185–187 °C (ethyl acetate). ^1^H NMR (400.1 MHz, CDCl_3_): δ 6.76 (d, *J* = 9.3 Hz, 1 H, CH-3), 7.38 (dd. *J* = 7.0, 1.3 Hz, 1 H, C_10_H_7_), 7.46–7.57 (m, 3H, C_10_H_7_), 7.60 (d, *J* = 2.5, 1H, CH-6), 7.70 (dd, *J* = 9.3, 2.5 Hz, 1H, CH-4), 7.83–7.97 (m, 3H, C_10_H_7_), 13.52 (br s, 1H, NH). ^13^C NMR (100.6 MHz, CDCl_3_): δ 119.58 (CH-3), 120.61 (=C-5), 125.06, 125.49, 126.16, 126.58, 127.00, 128.45, 128.60, 131.54, 133.91 (C_10_H_7_), 134.42 (CH-4), 134.70 (C_10_H_7_), 144.49 (CH-6), 164.86 (C-2). GC-MS (EI, 70 eV); *m*/*z* = 221 (100) [M^+^], 220 (25), 192 (37), 191 (16), 165 (44), 164 (14), 95 (13). HRMS (ESI-TOF): *m*/*z* calcd. for C_15_H_12_NO [M+H]^+^ 222.0919; found: 222.0917.

5-(Thiophen-2-yl)pyridin-2(1*H*)-one [**8**(**NH**)]: Yield 65%. Mp. 179–183 °C. ^1^H NMR (400 MHz, CDCl_3_) δ 6.67 (dd, *J* = 9.4, 0.7 Hz, 1H, CH-3), 7.05 (dd, *J* = 5.1, 3.6 Hz, 1H, CH-4′), 7.10 (dd, *J* = 3.6, 1.2 Hz, 1H, CH-3′), 7.24 (dd, *J* = 5.1, 1.2 Hz, 1H, CH-5′), 7.69 (dd, *J* = 2.7, 0.7 Hz, 1H, CH-6), 7.76 (dd, *J* = 9.4, 2.7 Hz, 1H, CH-4). ^13^C NMR (101 MHz, CDCl_3_) δ 115.78, (=C-5) 120.35 (CH-3), 122.70 (CH-3′), 124.36 (CH-5′), 128.11 (CH-4′), 131.07 (=CH-6), 139.27 (C-2′), 140.83 (CH-4), 164.65 (C=O). GC-MS (EI, 70 eV); *m*/*z* = 178 (11), [M^+^], 177 (100), 149 (39), 148 (12), 121 (23). HRMS (ESI-TOF): *m*/*z* calcd. for C_9_H_8_NOS [M+H]^+^ 178.0327; found: 178.0325.

**Method B:** A solution of 2-methoxy-5-arylpyridine (0.0106 mol), LiCl (2.24 g, 0.053 mol) and PTSA (9.1 g, 0.053 mol) in DMF (35 mL) was stirred at 120° C for 3 h. After this time, next portion of LiCl (2.97 g, 32.5 mmol) and PTSA (9.1 g, 0.053 mol) was added and the mixture was heated next 3 h at 120 °C. After cooling the reaction mixture was quenched by the addition of water (15 mL) and extracted with EtOAc (100 mL × 3) and washed with brine (15 mL × 3). The combined organic phase was dried over Na_2_SO_4_, filtered and concentrated. The residue was purified by column chromatography (SiO_2_, ethyl acetate).

5-(3-Methoxyphenyl)pyridin-2(1*H*)-one [69] [**5**(**NH**)]. Yield 80%. ^1^H and ^13^C NMR spectra matched those previously reported [69].

5-(3,4-Dimethoxyphenyl)pyridin-2(1*H*)-one [**6**(**NH**)]: Yield 71%. ^1^H NMR (400 MHz, CDCl_3_) δ 3.91 (s, 3H, OCH_3_), 3.93 (s, 3H, OCH_3_), 6.69 (d, *J* = 9.4 Hz, 1H, CH-3), 6.88–6.94 [m, 2H, C_6_H_3_(OCH_3_)_2_], 6.98 (dd, *J* = 8.3, 2.1 Hz, 1H, C_6_H_3_(OCH_3_)_2_), 7.62 (d, *J* = 2.6 Hz, 1H, CH-6), 7.78 (dd, *J* = 9.4, 2.6 Hz, 1H, CH-4), 13.86 (br. s, 1H, NH). ^13^C NMR (101 MHz, CDCl_3_) 55.97, 56.01 (two OCH_3_), 109.11, 111.74, 118.17 [C_6_H_3_(OCH_3_)_2_], 120.09 (CH-3), 121.30 (C-5), 129.27 [C_6_H_3_(OCH_3_)_2_], 131.52 (CH-6), 141.49 (CH-4), 148.69, 149.46 [C_6_H_3_(OCH_3_)_2_], 164.83 (C=O). GC-MS (EI, 70 eV); decomposition. HRMS (ESI-TOF): *m*/*z* calcd. for C_13_H_14_NO_3_ [M+H]^+^ 232.0974; found: 232.0978.

### Appendix A.2. Synthesis and Spectroscopic Data of Compounds **O1**, **O8**-**12**, **O18**-**22**

General procedure for the benzylation of 5-arylpyridones:

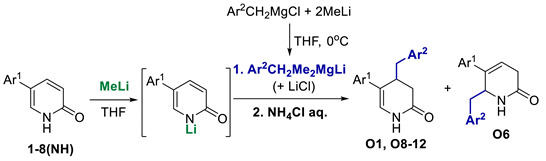

To 100 mL Schlenk flask the solution of BnMgCl (2.0 M in THF; 9.7 mmol, 4.85 mL) and dry THF (15 mL) was added under argon and cooled to 0 °C, followed by addition of MeLi (3.1 M in diethoxymethane; 19.4 mmol, 6.3 mL) with a syringe over 5 min. The resulting solution was stirred for 5 min at 0 °C. In a second Schlenk flask (250 mL), N-lithium 2-pyridone solution was prepared by adding with a syringe a solution of MeLi (3.1 M in diethoxymethane; 7.08 mmol, 2.29 mL) to 5-aryl-2-pyridone [**1**-**8**(**NH**)] solution (6.44 mmol) in THF (100 mL) at 0 °C. The solution prepared in the first Schlenk flask was then transferred to a cooled (0 °C) solution prepared in the second Schlenk flask. The resulting solution was stirred for 15 min at 0 °C and 120–180 min at rt (TLC or GC-MS control). After this time, the mixture was carefully quenched with saturated aqueous NH_4_Cl (30 mL). The aqueous layer was extracted with ethyl acetate (3 × 90 mL) and the combined organic layer were dried with MgSO_4_. The mixture was filtered, and the solvents were evaporated under reduced pressure. The crude product was purified by column chromatography on silica gel using appropriate solvents.

NOTE: BnMgCl (2.0 M in THF) was commercially available, while α-NaphCH_2_MgCl (0.05 M in THF) was prepared and titrated according to the procedure described earlier. [70]

*p*-MeOC_6_H_4_CH_2_MgCl in THF (**A**) and *p*-PhC_6_H_4_CH_2_MgCl in THF (**B**) were prepared as follows:

A Schlenk flask (100 mL) equipped with a 3 cm long stir bar was heated twice with a heat gun under argon. Then magnesium turnings (Sigma-Aldrich Merck Group, St. Louis, MO, USA) (**A**: 1.19 g, 0.0489 mol, **B**: 1.52 g, 0.0624 mol), porcelain finely broken (about half the volume of magnesium used) and iodine (catalytic amount) was added and heated again three short times, next vigorous stirring was started to grind the magnesium turnings (1 h at the speed of 600 rpm). Next dry THF (**A**: 42 mL **B**: 52 mL) was added (rt). In a second Schlenk flask the solution of [**A**: p-MeOC_6_H_4_CH_2_Cl (1.31 g, 8.87 mmol) in THF (5 mL), **B**: *p*-PhC_6_H_4_CH_2_Cl (2.1 g, 0.01 mol) in THF (7 mL)] was prepared, and the content of the second flask was added dropwise with a syringe to a suspension of Mg in THF over 120 min stirred at the speed of 500 rpm. The content of the flask was then allowed to stand for 30 min, and after this time a clear Grignard solution was transferred into separate Schlenk with a syringe from above the residue of Mg. The clear solution was titrated using a method described in the literature [71]. Grignard reagent **A** was obtained in 64% yield (0.13 M), **B** 29 % yield (0,06 M).

(4*RS*)-4-Benzyl-5-phenyl-3,4-dihydropyridin-2(1*H*)-one (**O1**) [15] Yield 62%. Spectra of compound **O1** are in agreement with literature data [15]. Note: In the step of lithation of 2-pyridone 1.7-fold excess was used and in this conditions apart from O1 also by-product **O6** was obtained.

(6*RS*)-6-Benzyl-5-phenyl-3,6-dihydropyridin-2(1*H*)-one (**O6**): The crude product purified by column chromatography (SiO_2_, ethyl acetate), gave colorless solid in 14% yield. Mp. 187–189 °C. ^1^H NMR (400 MHz, CDCl_3_): *δ* 2.50 (ddd, *J* = 22.2, 3.8, 2.7 Hz, 1 H, CHH-3), 2.63 (dd, *J* = 13.6, 7.2 Hz, 1 H, 6-CHH),), 2.90 (ddd, *J* = 22.2, 4.9, 3.0 Hz, 1H, CHH-3), 2.96 (dd, *J* = 13.6, 3.5 Hz, 1H, 6-CHH), 4.81 (dp, *J* = 7.2, 3.5 Hz, 1H, CH-6), 5.91 (dd, *J* = 4.9, 2.7 Hz, 1 H, =CH-4), 6.55–6.64 (br. s, 1 H, NH), 7.05–7.16 (m, 2 H, C_6_H_5_), 7.19–7.48 (m, 8H, C_6_H_5_). ^13^C NMR (100 MHz, CDCl_3_): *δ* 31.79 (CH_2_-3), 41.88 (6-CH_2_), 56.59 (CH-6), 120.99 (=CH-4), 126.29, 126.96, 128.03, 128.49, 128.92, 129.90 (two C_6_H_5_), 135.86 (=C-5), 136.10, 138.04 (two C_6_H_5_), 170.21 (C=O). GC-MS (EI, 70 eV): *m*/*z* = 263(3), [M^+^], 173 (12), 172 (100), 145 (10), 127 (11), 115 (16), 91 (16). HRMS (ESI-TOF): *m*/*z* calcd. for C_18_H_17_NNaO [M+Na]^+^ 286.1208; found: 286.1212.

(4*RS*)-4-Benzyl-5-(4-fluorophenyl)-3,4-dihydropyridin-2(1*H*)-one (**O8**): The crude product purified by column chromatography (SiO_2_, *n*-hexane: ethyl acetate 1:1), gave white solid in 72% yield. Mp. 124–125 °C (petroleum ether:ethyl acetate). ^1^H NMR (400.1 MHz, CDCl_3_): δ 2.51 (ddd, *J* = 16.5, 2.1, 1.0 Hz, 1H, CHH-3), 2.58 (dd, *J* = 10.7, 6.3 Hz, 1H, 4-CHH), 2.63 (ddd, *J* = 16.2, 6.5, 0.6 Hz, 1H, CHH-3), 2.86 (dd, *J* = 13.7, 4.2 Hz, 1H, 4-CHH), 3.08 (dddd, *J* = 10.7, 6.5, 4.2, 2.2 Hz, 1H, CH-4), 6.47 (d, *J* = 4.8 Hz, 1H, =CH-6), 7.00–7.12 (m, 2 H, C_6_H_5_), 7.14–7.24 (m, 3 H, C_6_H_5_), 7.24–7.41 (m, 4 H, C_6_H_5_), 8.13 (br s, 1 H, NH). ^13^C NMR (100.6 MHz, CDCl_3_): δ 34.19 (CH_2_-3), 36.71 (CH-4), 37.86 (4-CH_2_), 115.69 (d, ^2^*J*_C-F_ = 21.6 Hz, CH-3′, CH-5′), 120.74 (=C-5), 120.91 (=CH-6), 126.53 (C_6_H_5_), 126.55 (d, ^3^*J*_C-F_ = 7.5 Hz, CH-2′, CH-6′) 126.50, 129.38, (C_6_H_5_), 133.33 (d, ^4^*J*_C-F_ = 3.3 Hz, C-1′), 138.47 (C_6_H_5_), 161.82 (d, ^1^*J*_C-F_ = 246.6 Hz, C-4′), 170.52 (C=O). GC-MS (EI, 70 eV); *m*/*z* = 281 (11), [M^+^], 191 (13), 190 (100), 163 (18), 145 (9), 133 (20), 115 (15), 91 (28), 65 (12). Anal. calcd for C_18_H_16_FNO: C 76.85, H 5.73, N 4.98. Found: C 76.86, H 5.75, N 5.06.

(4*RS*)-4-Benzyl-5-(3,4-difluorophenyl)-3,4-dihydropyridin-2(1*H*)-one (**O9**): The crude product purified by column chromatography (SiO_2_, *n*-hexane: ethyl acetate 1:1), gave colorless semisolid in 41% yield. ^1^H NMR (400 MHz, CDCl_3_): δ 2.46–2.71 (m, 3H, CH_2_-3, 4-CHH), 2.85 (dd, *J* = 13.7, 4.6 Hz, 1H, 4-CHH), 2.98–3.13 (m, 1H, CH-4), 6.50 (d, *J* = 4.8 Hz, 1H, CH-6), 7.03–7.35 (m, 8H, C_6_H_5_, C_6_H_3_F_2_), 8.27 (br s, 1H, NH). ^13^C NMR (101 MHz, CDCl_3_): δ 34.25(CH_2_-3), 36.54 (CH-4), 37.94 (4-CH_2_), 113.84 (d, *J* = 18.0 Hz, CH-2′), 117.54 (d, *J* = 17.4 Hz, CH-5′), 119.76 (C-5), 120.82 (dd, *J* = 6.1, 3.5 Hz, CH-6′), 121.86 (CH-6), 126.65, 128.55, 129.38 (C_6_H_5_), 134.52 (dd, *J* = 6.4, 3.9 Hz, C-1′), 138.23 (C_6_H_5_), 149.19 (dd, *J* = 248.8, 12.8 Hz, C-3′), 150.50 (dd, *J* = 247.3, 12.8 Hz, C-4′), 170.54 (C=O). GC-MS (EI, 70 eV); *m*/*z* = 299 (8), [M^+^], 208 (100), 207 (42), 179 (18), 151 (21), 133 (23), 91 (43), 65 (18). HRMS (ESI-TOF): *m*/*z* calcd. for C_18_H_15_NNaO [M+Na]^+^ 322.1019; found: 322.1027.

(4*RS*)-4-Benzyl-5-(4-chlorophenyl)-3,4-dihydropyridin-2(1*H*)-one (**O10**): The crude product purified by column chromatography (SiO_2_, *n*-hexane: ethyl acetate 3:1), gave white solid in 65% yield. Mp. 139–142 °C (petroleum ether: ethyl acetate). ^1^H NMR (400 MHz, CDCl_3_): δ 2.51 (dt, *J* = 16.4, 1.3 Hz, 1H, CHH-3), 2.57–2.64 (m, 2H, 4-CHH, CHH-3), 2.86 (dd, *J* = 13.7, 4.1 Hz, 1H, 4-CHH), 3.05–3.09 (m, 1H, CH-4), 6.55 (d, *J* = 4,8 Hz, 1H, CH-6), 7.16–7.31 (m, 9H, C_6_H_5_, C_6_H_4_), 8.61 (d, *J* = 4.8, 1H, NH). ^13^C NMR (100 MHz, CDCl_3_): δ 34.13 (CH_2-_3), 36.32 (CH-4), 37.86 (4-CH_2_), 120.33 (=C-5), 121.53 (=CH-6), 126.08, 126.56, 128.52, 128.9, 129.38, 132.46, 135.66, 138.39 (C_6_H_5_, C_6_H_4_), 198.1 (C=O). GC-MS (EI, 70 eV): *m*/*z* = 297 (9), [M^+^], 208 (33), 206 (100), 205 (9), 144 (14), 143 (27), 115 (21), 91 (17). Anal. calcd for C_18_H_16_ClNO: C 72.60, H 5.42, N 4.70. Found: C 72.73, H 5.35, N 4.81.

(4*RS*)-4-Benzyl-5-(3-methoxyphenyl)-3,4-dihydropyridin-2(1*H*)-one (**O11**): The crude product purified by column chromatography (SiO_2_, *n*-hexane: ethyl acetate 1:1), gave colorless solid in 65% yield. Mp. 133–135 °C (petroleum ether: ethyl acetate). ^1^H NMR (400 MHz, CDCl_3_): = δ 2.51 (ddd, *J* = 16.5, 2.1, 1.0 Hz, 1H, CHH-3), 2.56–2.67 (m, 2H, CHH-3, 4-CHH), 2.91 (dd, *J* = 13.7, 3.9 Hz, 1H, 4-CHH), 3.11 (dddd, *J* = 10.6, 6.5, 3.9, 2.1 Hz, 1H, CH-4), 3.84 (s, 3H, OCH_3_), 6.56 (d, *J* = 4.8 Hz, 1H, =CH-6), 6.81 (ddd, *J* = 8.2, 2.6, 0.9 Hz, 1H, C_6_H_4_), 6.95 (dd, *J* = 2.5, 1.7 Hz, 1H, C_6_H_4_), 7.02 (ddd, *J* = 7.7, 1.8, 0.9 Hz, 1H, C_6_H_4_), 7.16–7.35 (m, 6H, C_6_H_4_, C_6_H_5_, 8.06 (br s, 1H, NH). ^13^C NMR (100 MHz, CDCl_3_) δ 34.06 (CH_2_-3), 36.45 (CH-4), 37.88 (4-CH_2_), 55.27 (OCH_3_), 110.95, 111.93, 117.38 (C_6_H_4_), 121.26 (=CH-6), 121.30 (=C-5), 126.48, 128.49, 129.43, 129.81, 138.62 (overlapped signals), 159.99 (C_6_H_4_, C_6_H_5_), 170.59 (C=O); GC-MS (EI, 70 eV): *m*/*z* = 293(13), [M+], 203 (15), 202 (100), 159 (8), 130 (8), 91 (12). HRMS (ESI-TOF): *m*/*z* calcd. for C_19_H_19_NNaO_2_ [M+Na]^+^ 316.1313; found: 316.1319.

(4*RS*)-4-Benzyl-5-(3,4-dimethoxyphenyl)-3,4-dihydropyridin-2(1*H*)-one (**O12**). The crude product purified by column chromatography (SiO_2_, *n*-hexane: ethyl acetate 1:1), gave white solid in 61% yield. Mp. 164–166 °C (petroleum ether: ethyl acetate). ^1^H NMR (400 MHz, CDCl_3_): δ 2.50 (ddd, *J* = 16.5, 2.0, 1.0 Hz, 1H, CHH-3), 2.56–2.68 (m, 2H, CHH-3, 4-CHH), 2.89 (dd, *J* = 13.7, 3.9 Hz, 1H, 4-CHH), 3.08 (qd, *J* = 6.6, 5.2, 2.0 Hz, 1H, CH-4), 3.90 and 3.91 (two s, 6H, two OCH_3_), 6.50 (d, *J* = 4,7 Hz, 1H, =CH-6), 6.85–6.92 (m, 2H, C_6_H_3_), 6.98 (dd, *J* = 8.3, 2.2 Hz, 1H, C_6_H_3_), 7.14–7.36 (m, 5H, C_6_H_5_)_,_ 8.64 (d, *J* = 4.7 Hz, 1H, NH). ^13^C NMR (100 MHz, CDCl_3_): δ 34.15 (CH_2_-3), 36.69 (CH-4), 37.90 (4-CH_2_), 55.91 and 55.96 (two OCH_3_), 108.31, 111.40, 117.40 (C_6_H_3_), 119.96 (=CH-6), 121.49 (=C-5), 126.46, 128.49, 129.39, 130.07, 138.79, 148.26, 149.14 (C_6_H_3_, C_6_H_5_), 170.95 (C=O). GC-MS (EI, 70 eV); *m*/*z* = 323 (23), [M^+^], 233 (15), 232 (100), 207 (11), 91 (17). Anal. calcd for C_20_H_21_NO_3_: C 74.28, H 6.55, N 4.33. Found: C 74.34, H 6.73, N 4.50.

4-(Naphthalen-2-ylmethyl)-5-phenyl-3,4-dihydropyridin-2(1*H*)-one (**O18**) was obtained with 5% of binaphtyl, which could not be removed and contaminated **O18** was submitted for thionation.

(4*RS*)-4-(4-Methoxybenzyl)-5-phenyl-3,4-dihydropyridin-2(1*H*)-one (**O19**): The crude product purified by column chromatography (SiO_2_, *n*-hexane: ethyl acetate 1:1) gave colorless solid in 88% yield. Mp. 149–152 °C (petroleum ether: ethyl acetate). ^1^H NMR NMR (400 MHz, CDCl_3_): δ 2.44–2.67 (m, 3H, CH_2_-3, 4-CHH), 2.84 (dd, *J* = 13.9, 3.8 Hz, 1H, 4-CHH), 3.02–3.14 (m, 1H, CH-4), 3.77 (s, 3H, OCH_3_), 6.55 (d, *J* = 4.4 Hz, 1H, =CH-6), 6.78 (m, 2H, C_6_H_4_), 7.05–7.17 (m, 2H, C_6_H_4_), 7.26 (tt, *J* = 7.0, 1.5 Hz, 1H, C_6_H_5_), 7.33–7.47 (m, 4H, C_6_H_5_), 8.37 (br s, 1H, NH). ^13^C NMR (100 MHz, CDCl_3_): δ 34.01 (CH_2_-3), 36.48 (CH-4), 36.95 (4-CH_2_), 55.25 (O-CH_3_), 113.87 (2C, C_6_H_4_), 120.96 (=CH-6), 121.53 (=C-5), 124.82, 126.83, 128.83 (C_6_H_5_), 130.36 (2C), 130.65 (C_6_H_4_), 137.14, (C_6_H_5_), 158.26 (C_6_H_4_), 170.91 (C=O). GC-MS (EI, 70 eV); *m*/*z* = 293 (11) [M^+^], 172 (55), 171 (21), 121 (100), 115 (11). Anal. calcd for C_19_H_19_NO_2_: C 77.79, H 6.53, N 4.77. Found: C 77.64, H 6.73, N 4.95.

(4*RS*)-4-([1,1′-biphenyl]-4-ylmethyl)-5-phenyl-3,4-dihydropyridin-2(1*H*)-one (**O20**): The crude product purified by column chromatography (SiO_2_, *n*-hexane: ethyl acetate 1:1) gave colorless solid in 48% yield. Mp. 207–209 °C (petroleum ether: ethyl acetate). ^1^H NMR (400 MHz, CDCl_3_): δ 2.52–2.70 (m, 3H, CH_2_-3, 4-CHH), 2.94 (dd, *J* = 13.8, 3.9, 1H, 4-CHH), 3.11–3.21 (m, 1H, CH-4) 6.56 (d, *J* = 4.8 Hz,1H, =CH-6), 7.23–7.47 m, 10H, ArH), 7.49–7.61 (m, 4H, ArH), 7.98 (br. s, 1H, NH). ^13^C NMR (100 MHz, CDCl_3_): δ 34.15 (CH_2_-3), 36.41 (CH-4), 37.50 (4-CH_2_), 121.01 (=CH-6), 121.48 (=C-5), 124.86 (2C), 126.88, 127.00 (2C), 127.14, 127.18 (2C), 128.74 (2C), 128.85 (2C), 129.84 (2C), 137.09, 137.71, 139.40, 140.89 (ArH), 170.52 (C=O). GC-MS (EI, 70 eV): *m*/*z* = 339 (5) [M^+^], 172 (100), 167 (22), 165 (11), 115 (10). Anal. calcd for C_24_H_21_NO: C 84.92, H 6.24, N 4.13. Found: C 84.62, H 6.16, N 4.21.

(4*RS*)-4-Benzyl-5-(naphthalen-1-yl)-3,4-dihydropyridin-2(1*H*)-one (**O21**): The crude product purified by column chromatography (SiO_2_, *n*-hexane: ethyl acetate 3:1) gave colorless solid in 41% yield. Mp. 174–76 °C (ethyl acetate). ^1^H NMR NMR (400 MHz, CDCl_3_): δ 2.49–2.64 (m, 2H, CHH-3, 4-CHH), 2.76–2.90 (m, 2H, CHH-3, 4-CHH), 3.04 (ddt, *J* = 10.8, 7.1, 3.6 Hz, 1H, CH-4), 6.25 (d, *J* = 4.6 Hz, 1H, =CH-6), 6.87–7.06 (m, 2H, ArH), 7.07–7.22 (m, 3H, ArH), 7.35 (dd, *J* = 7.0, 1.3 Hz, 1H, ArH), 7.40–7.56 (m, 3H, ArH), 7.83 (dt, *J* = 8.2, 1.0 Hz, 1H, ArH), 7.87–7.93 (m, 1H, ArH), 7.98 (dd, *J* = 6.2, 3.5 Hz, 1H, ArH), 8.38 (d, *J* = 4.6 Hz, 1H, NH).^13^C NMR (100 MHz, CDCl_3_): δ 34.29 (CH_2_-3), 37.59 (4-CH_2_), 39.51 (CH-4), 121.95 (=C-5), 123.61 (=CH-6), 125.32, 125.42, 125.94, 126.24, 126.30, 127.00, 128.01, 128.38 (2C), 128.62, 129.27 (2C), 132.23, 133.90, 136.51, 138.58, (C_10_H_7_, C_6_H_5_) 170.73 (C=O). GC-MS (EI, 70 eV); *m*/*z* = 313 (15) [M^.+^], 222 (100), 205 (14), 193 (10), 167 (12), 165 (25), 152 (11), 91 (13). HRMS (ESI-TOF): *m*/*z* calcd. for C_22_H_20_NO [M+H]^+^ 314.1545; found: 314.1546.

(4*RS*)-4-benzyl-5-(thiophen-2-yl)-3,4-dihydropyridin-2(1*H*)-one (**O22**): The crude product purified by column chromatography (SiO_2_, *n*-hexane: ethyl acetate 2:1) gave white solid in 59% yield. Mp. 137–139 °C (hexane: ethyl acetate). ^1^H NMR (400 MHz, CDCl_3_) δ 2.50 (dt, *J* = 16.5, 1.5 Hz, 1H, CHH-3), 2.58–2.68 (m, 2H, CHH-3, 4-CHH), 2.97 (dd, *J* = 13.5, 3.9 Hz, 1H, 4-CHH), 3.04 (dddd, *J* = 10.5, 6.5, 3.9, 1.5 Hz, 1H, CH-4), 6.58 (d, *J* = 4.8 Hz, 1H, =CH-6), 6.98 (dd, *J* = 3.6, 1.2 Hz, 1H, C_4_H_3_S), 7.01 (dd, *J* = 5.1, 3.6 Hz, 1H, C_4_H_3_S), 7.16 (dd, *J* = 5.0, 1.2 Hz, 1H, C_4_H_3_S), 7.19–7.25 (m, 3H, C_6_H_5_), 7.31 (ddt, *J* = 7.7, 6.3, 1.2 Hz, 2H, C_6_H_5_), 7.90 (d, *J* = 4.8 Hz, 1H, NH). ^13^C NMR (101 MHz, CDCl_3_) δ 33.92 (CH_2_-3), 37.89 (CH-4), 37.99 (4-CH_2_), 116.75 (=C-5), 119.98 (=CH-6), 121.89 (C_4_H_3_S), 123.07 (C_4_H_3_S), 126.57 (C_6_H_5_), 127.71 (C_4_H_3_S), 128.53 (2C, C_6_H_5_), 129.48 (2C, C_6_H_5_), 138.48 (C_6_H_5_), 141.56 (C_4_H_3_S), 170.18 (C=O). GC-MS (EI, 70 eV); *m*/*z* = 269 (54), [M^+^], 179 (19), 178 (100), 177 (14), 151 (22), 149 (11), 117 (17), 91 (16). HRMS (ESI-TOF): *m*/*z* calcd. for C_16_H_15_NNaOS [M+Na]^+^ 292.0772; found: 292.0772.

### Appendix A.3. Synthesis and Spectroscopic Data of N-alkylated **O2**, **O3**, **O7**, **O13**-**17**

General procedure for the N-alkylation of NH, 4-benzyl-3,4-aryl-3,4-dihydropyridin-2(1*H*)-ones **O2**, **O3**, **O13**-**17**

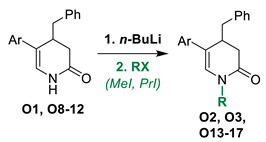

The solution of 5-aryl-4-benzyl-3,4-dihydropyridin-2(1*H*)-one (**O1**, **O8**-**12**) (1.82 mmol) in dry THF (30 mL) was placed in a 50 mL Schlenk flask under argon. To the solution a portion of *n*-BuLi (2.5 M in hexane, 2.0 mmol, 0.8 mL) was slowly added under argon with a syringe at 0 °C under stirring. The mixture was stirred 5 min at 0 °C, then MeI (4.56 mmol, 0.647 g) or PrI (2.55 mmol, 0.433 g) was added with a syringe. The resulting solution was warmed up to rt and stirred for 2 h (MeI) or 48h (PrI). After this time saturated aqueous NH_4_Cl (10 mL) was added and the aqueous layer was extracted with ethyl acetate (3 × 60 mL), and the combined organic layers were dried with MgSO_4_. The mixture was filtered, and the solvents were evaporated under reduced pressure. The crude product was purified by column chromatography on SiO_2_ using appropriate solvent as eluent.

4-Benzyl-1-methyl-5-phenyl-3,4-dihydropyridin-2(1*H*)-one (**O2**): (From **O1**.) The crude product purified by column chromatography (SiO_2_, *n*-hexane: ethyl acetate, 3: 1), gave white solid in 79% yield. Mp. 86–88 °C. ^1^H NMR (400 MHz, CDCl_3_): δ 2.51–2.65 (m, 3H, CH_2–_3, 4-CHH), 2.87 (dd, *J* = 13.7, 3.9 Hz, 1H, 4-CHH), 3.07 (s, 3H, NCH_3_), 3.08 (m, CH-4), 6.44 (d, *J* = 0.6 Hz, 1H, =CH-6), 7.15–7.22 (m, 3H, C_6_H_5_), 7.23–7.32 (m, 3H, C_6_H_5_), 7.34–7.44 (m, 4H, C_6_H_5_). ^13^C NMR (101 MHz, CDCl_3_): δ 33.90 (CH-4), 34.97 (CH_2–_3), 36.51 (NCH_3_), 38.22 (4-CH_2_), 121.82 (=C-5), 124.80, 126.44 126.82, (C_6_H_5_), 127.12 (=CH-6), 128.34, 128.80, 129.46, 137.11, 138.53 (C_6_H_5_), 168.41 (C=O). GC-MS (EI, 70 eV): *m*/*z* = 277 (7), [M+], 187 (13), 186 (100), 158 (5), 143 (11), 115 (11), 91 (10). HRMS (ESI-TOF): *m*/*z* calcd. for C_19_H_19_NNaO [M+Na]^+^ 300.1364; found: 300.1371.

4-Benzyl-5-phenyl-1-propyl-3,4-dihydropyridin-2(1*H*)-one (**O3**): (From **O1**.) The crude product purified by column chromatography (SiO_2_, *n*-hexane: ethyl acetate, 3: 1), gave pale yellow oil in 37% yield. ^1^H NMR (400 MHz, CDCl_3_): δ 1.01 (t, *J* = 7.4 Hz, 3H, CH_3_), 1.61 (h, *J* = 7.4 Hz, 2H, CH_2_), 2.48–2.67 (m, 3H, 4-CHH, CH_2_-3) 2.88 (dd, *J* = 13.6, 4.0 Hz, 1H, 4-CHH), 3.00–3.13 (m, 1H, CH-4) 3.28 (dt, *J* = 13.5, 7.3 Hz, 1H, NCHH), 3.69 (dt, *J* = 13.4, 7.4 Hz, 1H, NCHH), 6.47 (d, *J* = 0.6 Hz, 1H, =CH-6), 7.11–7.31 (m, 6H, C_6_H_5_), 7.33–7.45 (m, 4H, C_6_H_5_). ^13^C NMR (101 MHz, Chloroform) *δ* (ppm) 11.35 (CH_3_), 22.08 (CH_2_), 35.10 (CH_2_-3), 36.47 (CH-4), 38.05 (4-CH_2_), 48.06 (NCH_2_), 121.88 (=C-5), 124.77 (C_6_H_5_), 126.21 (=CH-6), 126.42, 126.75, 128.42, 128.81, 129.44, 137.29, 138.68 (two C_6_H_5_), 168.03 (C=O). GC-MS (EI, 70 eV): *m/z* = 305 (8), [M^+^], 215 (17), 214 (100), 172 (36), 115 (10), 91 (12). HRMS (ESI-TOF): *m*/*z* calcd. for C_21_H_23_NNaO [M+Na]^+^ 328.1677; found: 328.1673.

(4*RS*)-4-Benzyl-5-(4-fluorophenyl)-1-methyl-3,4-dihydropyridin-2(1*H*)-one (**O13**). (From **O8**.) The crude product purified by column chromatography (SiO_2_, *n*-hexane: ethyl acetate 1:1), gave white solid in 90% yield. Mp. 110–112 °C (petroleum ether: ethyl acetate). ^1^H NMR (400.1 MHz, CDCl_3_): δ 2.52–2.62 (m, 3H, CH_2_-3, 4-CHH), 2.82 (dd, *J* = 13.6, 4.2, 1H, 4-CHH), 3.01–3.07 (m, 1H, CH-4), 3.07 (s, 3H, NCH_3_), 6.36 (s, 1H, =CH-6), 7.00–7.09 (m, 2 H, C_6_H_5_), 7.12–7.17 (m, 2 H, C_6_H_5_), 7.17–7.24 (m, 2 H, C_6_H_5_), 7.25–7.31 (m, 1H, C_6_H_5_), 7.33–7.39 (m, 2H, C_6_H_5_). ^13^C NMR (100.6 MHz, CDCl_3_): δ 33.88 (CH-4), 35.06 (CH_2_-3), 36.83 (NCH_3_), 38.22 (4-CH_2_), 115.68 (d, ^2^*J*_C-F_ = 21.5 Hz, CH-3′, CH-5′), 121.09 (=C-5), 126.52 (d, ^3^*J*_C-F_ = 7.8 Hz, CH-2′, CH-6′), 126.55 (C_6_H_5_), 127 (=CH-6), 128.35, 129.41 (C_6_H_5_), 133.34 (d, ^4^*J*_C-F_ = 3.4 Hz, C-1′), 138.37 (C_6_H_5_), 161.81 (d, ^1^*J*_C-F_ = 246.5 Hz, C-4′), 168.25 (C=O). GC-MS (EI, 70 eV); *m*/*z* = 295 (7), [M^+^], 205 (13), 204 (100), 176 (9), 161 (13). HRMS (ESI-TOF): *m*/*z* calcd. for C_19_H_18_NNaO [M+Na]^+^ 318.127; found: 318.1262.

(4*RS*)-4-Benzyl-5-(3,4-difluorophenyl)-1-methyl-3,4-dihydropyridin-2(1*H*)-one (**O14**): (From **O9**.) The crude product purified by column chromatography (SiO_2_, *n*-hexane: ethyl acetate) gave white solid in 96% yield. Mp 89–91 °C (petroleum ether: ethyl acetate). ^1^H NMR (400 MHz, CDCl_3_): δ 2.52–2.66 (m, 3H, CH_2_-3, 4-CHH), 2.81 (dd, *J* = 13.6, 4.4 Hz, 1H, 4-CHH), 2.96–3.05 (m, 1H, CH-4), 3.07 (s, 3H, N-CH_3_), 6.39 (s, 1H, CH-6), 6.99–7.43 (m, 8H, C_6_H_5_, C_6_H_3_). ^13^C NMR (101 MHz, CDCl_3_): δ 33.97 (NCH_3_), 35.10 (CH_2_-3), 36.65 (CH-4), 38.29 (4-CH_2_), 113.75 (d, *J* = 18.0 Hz, CH-2′), 117.51 (d, *J* = 17.4 Hz, CH-5′), 120.01 (=C-5), 120.76 (dd, *J* = 5.8, 3.6 Hz, CH-6′), 126.62 (=CH-6), 127.89, 128.39, 129.39 (C_6_H_5_), 134.53 (dd, *J* = 6.6, 3.8 Hz, C-1′), 138.13 (C_6_H_5_), 149.16 (dd, *J* = 248.8, 12.6 Hz, C-3′), 151.65 (dd, *J* = 247.5, 13.2 Hz, C-4′), 168.22 (C=O). GC-MS (EI, 70 eV): *m*/*z* = 313 (6) [M+], 222 (100), 194 (11) 179 (16), 151 (8), 91 (7). Anal. calcd for C_19_H_17_F_2_NO: C 72.83, H 5.47, N 4.47. Found: C 73.09, H 5.75, N 4.52.

(4*RS*)-4-Benzyl-5-(4-chlorophenyl)-1-methyl-3,4-dihydropyridin-2(1*H*)-one (**O15**): (From **O10**.) The crude product purified by column chromatography (SiO_2_, *n*-hexane: ethyl acetate 1:1) gave white solid in 71% yield. Mp. 100–101 °C (petroleum ether: ethyl acetate). ^1^H NMR (400 MHz, CDCl_3_): δ 2.53–2.60 (m, 3H, 4-CHH, CH_2_-3), 2.82 (dd, *J* = 13.6, 4.1 Hz, 1H, 4-CHH), 3.01–3.06 (m, 1H, CH-4), 3.06 (s, 3H, NCH_3_), 6.43 (s, 1H, CH-6), 7.13–7.32 (m, 9H, C_6_H_5_, C_6_H_4_). ^13^C NMR (100 MHz, CDCl_3_): δ 33.94 (CH-4), 35.01 (CH_2_-3), 36.48 (NCH_3_), 38.24 (4-CH_2_), 120.67 (=C-5), 126.03, 126.53 (C_6_H_4_, C_6_H_5_), 127.51 (=CH-6), 128.36, 128.91, 129.40, 132.40, 132.42, 135.66, 138.27 (C_6_H_4_, C_6_H_5_), 168.28 (C=O). GC-MS (EI, 70 eV); *m*/*z* = 311 (8), [M^+^], 222 (33), 220 (100), 185 (6), 157 (17), 156 (6), 115 (11), 91 (8). HRMS (ESI-TOF): *m*/*z* calcd. for C_19_H_18_ClNNaO [M+Na]^+^ 334.0975; found: 334.0972.

(4*RS*)-4-Benzyl-5-(3-methoxyphenyl)-1-methyl-3,4-dihydropyridin-2(1*H*)-one (**O16**): (From **O11**.) The crude product purified by column chromatography (SiO_2_, *n*-hexane: ethyl acetate 1:1, gave pale yellow oil in 96% yield. ^1^H NMR (400 MHz, CDCl_3_) δ 2.49–2.67 (m, 3H, CH_2_-3, 4-CHH), 2.87 (dd, *J* = 13.6, 3.8 Hz, 1H, 4-CHH), 3.04–3.11 (m, 1H, CH-4), 3.06 (s, 3H, NCH_3_), 3.85 (s, 3H, OCH_3_), 6.45 (s, 1H, =CH-6), 6.81 (ddd, *J* = 8.2, 2.5, 0.8 Hz, 1H, C_6_H_4_), 6.95 (t, *J* = 2.1 Hz, 1H, C_6_H_4_), 7.03 (dt, *J* = 7.7, 1.8, 0.9 Hz, 1H, C_6_H_4_), 7.14–7.24 (m, 3H, C_6_H_4_, C_6_H_5_), 7.24–7.33 (m, 3H, C_6_H_5_). ^13^C NMR (101 MHz, CDCl_3_) δ 33.91 (CH-4), 34.95(CH_2_-3), 36.57 (NCH_3_), 38.26 (4-CH_2_), 55.29 (OMe), 111.03, 111.74, 117.35 (C_6_H_4_), 121.62 (=C-5), 126.45 (C_6_H_5_), 127.38 (=CH-6), 128.33, 129.46, 129.79, 138.50, 138.64, 159.99 (C_6_H_4,_ C_6_H_5_), 168.46 (C=O). GC-MS (EI, 70 eV): *m*/*z* = 307 (11), [M+], 217 (16), 216 (100), 173 (6), 144 (6), 115 (6) 91 (8). HRMS (ESI-TOF): *m*/*z* calcd. for C_20_H_21_NNaO_2_ [M+Na]^+^ 330.1470; found: 330.1465.

(4*RS*)-4-Benzyl-5-(3,4-dimethoxyphenyl)-1-methyl-3,4-dihydropyridin-2(1*H*)-one (**O17**): (From **O12**.) The crude product purified by column chromatography (SiO_2_, *n*-hexane: ethyl acetate 3:1) gave white solid in 91% yield. Mp. 109–110 °C (petroleum ether:ethyl acetate). ^1^H NMR (400 MHz, CDCl_3_): δ 2.52–2.66 (m, 3H, 4-CHH, CH_2_-3), 2.85 (dd, *J* = 13.6, 4.0, 1H, 4-CHH), 3.01–3.07 (m, 1H, CH-4), 3.09 (s, 3H, N-CH_3_), 3.91 (s, 3H, O-CH_3_), 3.92 (s, 3H, OCH_3_), 6.33–6.37 (m, 1H, =CH-6), 6.85–6.91 (m, 2H, C_6_H_5_), 6.98 (dd, *J* = 8.4, 2.2 Hz, 1H, C_6_H_5_), 7.14–7.24 (m, 3H, C_6_H_5_)_,_ 7.25–7.33 (m, 2H C_6_H_5_). ^13^C NMR (100 MHz, CDCl_3_): δ 33.88 (CH-4), 35.05 (CH_2_-3), 36.95 (N-CH_3_), 38.31 (4-CH_2_), 55.98 (2 × O-CH_3_), 108.40, 111.43, 117.51 (C_6_H_5_), 122.04 (=C-5), 125.97 (=CH-6), 126.46, 128.38, 129.44, 130.12, 138.68, 148.31, 149.16 (C_6_H_5_), 168.35 (C=O). GC-MS (EI, 70 eV); *m*/*z* = 337 (18) [M^+^], 247 (16), 246 (100), 205 (9), 91 (10). Anal. calcd for C_21_H_23_NO_3_: C 74.75, H 6.87, N 4.15. Found: C 74.67, H 7.06, N 4.35.

1,4-Dibenzyl-5-phenyl-3,4-dihydropyridin-2(1*H*)-one [15](**O4**): Prepared according to the procedure described earlier [15].

6-Benzyl-1-methyl-5-phenyl-3,6-dihydropyridin-2(1*H*)-one (**O7**): Prepared according to the procedure described earlier [15]. The crude product purified by column chromatography (SiO_2_, *n*-hexane: ethyl acetate 3:1), gave light cream semisolid in 82 % yield. ^1^H NMR (400 MHz, CDCl_3_): *δ* 1.69 (dt, *J* = 21.5,2.9 Hz, 1 H, CHH-3), 2.66–2.72 (m, 2 H, CHH-3, 6-CHH), 3.09 (dd, *J* = 13.8, 4.6 Hz, 1H, 6-CHH), 3.19 (s, 3H, NCH_3_), 4.61 (dtd, *J* = 4.9, 3.4, 1.6 Hz 1 H, CH-6), 5.86 (dd, *J* = 5.5, 2.4 Hz, 1H, =CH-4), 6.87–7.02 (m, 2 H, C_6_H_5_), 7.09–7.60 (m, 8H, C_6_H_5_). ^13^C NMR (100 MHz, CDCl_3_) *δ* = 33.25 (CH_2_-3), 33.56 (NCH_3_), 36.76 (6-CH_2_), 62.95 (CH-6), 122.12 (=CH-4), 126.18, 127.00, 128.00, 128.06, 128.93, 130.21 (C_6_H_5_), 135.21 (=C-5), 135.51, 137.71 (C_6_H_5_), 169.01 (C=O). GC-MS (EI, 70 eV): *m*/*z* = 277 (2), [M^+^], 187 (13), 186 (100), 158, 143 (11), 115 (13), 91 (13). HRMS (ESI-TOF): *m*/*z* calcd. for C_19_H_19_NNaO [M+Na]^+^ 300.1364; found: 300.1368.
