# Supplementary figures and images for: Synthesis and Anticancer Activity of Mitotic-Specific 3,4-Dihydropyridine-2(1H)-thiones
Source: Int J Mol Sci. 2021 Feb 28;22(5):2462. doi: 10.3390/ijms22052462 (PMC7957618; doi:10.3390/ijms22052462)

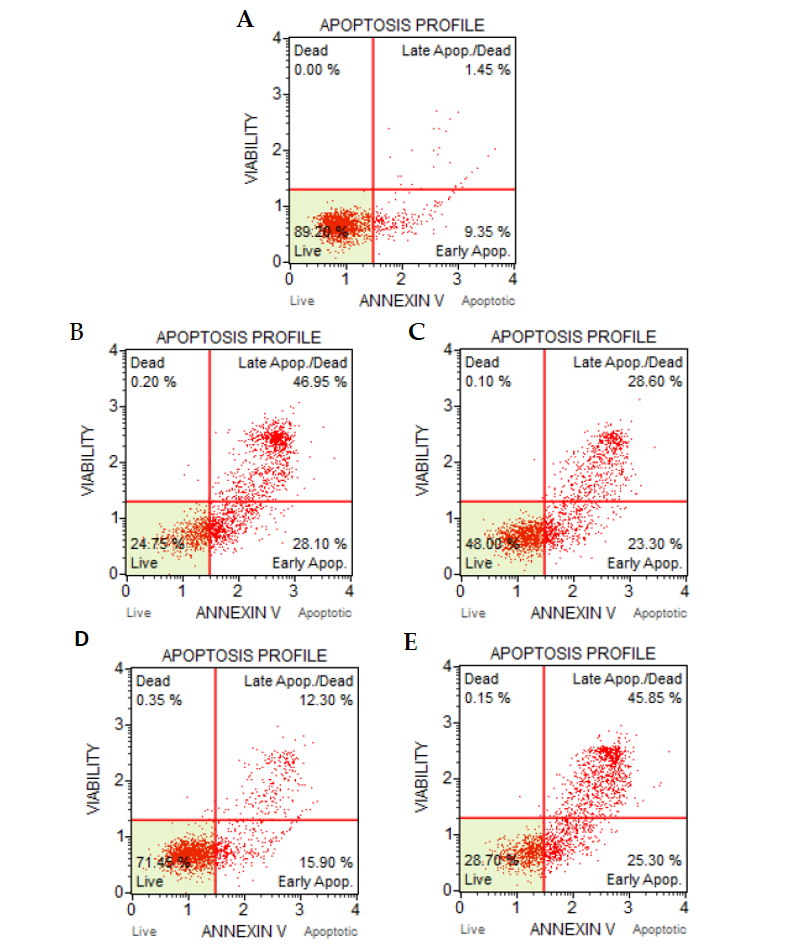

Supplement: Supplementary file 1 [file ijms-22-02462-s001.zip › Supplementary Materials/Figure S1.tif]

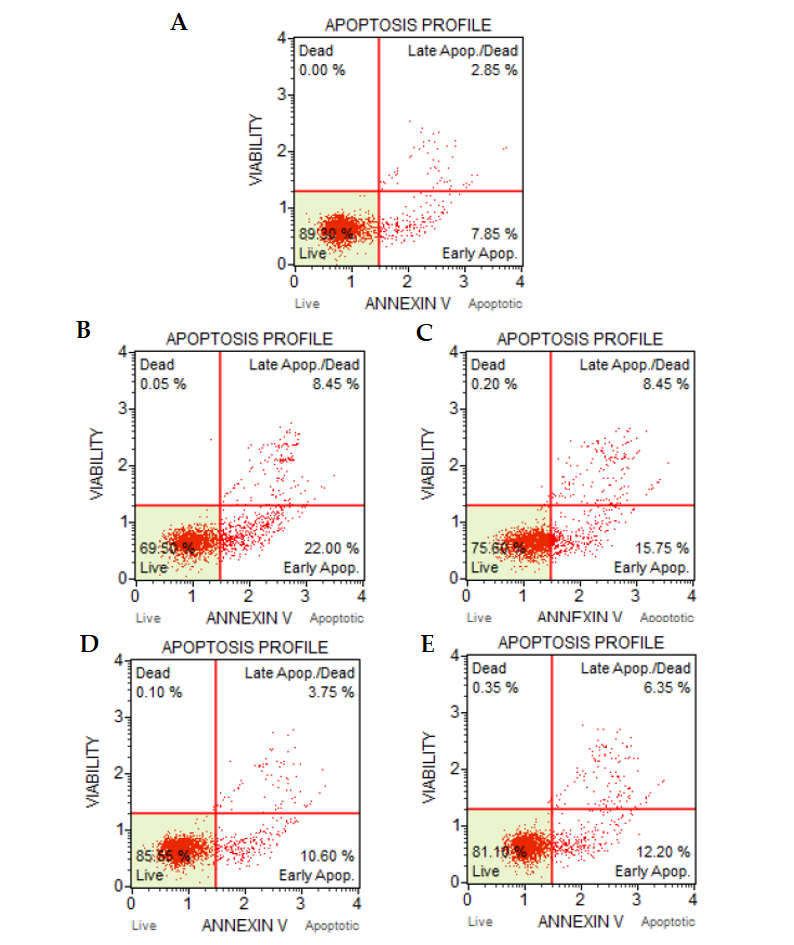

Supplement: Supplementary file 1 [file ijms-22-02462-s001.zip › Supplementary Materials/Figure S2.tif]

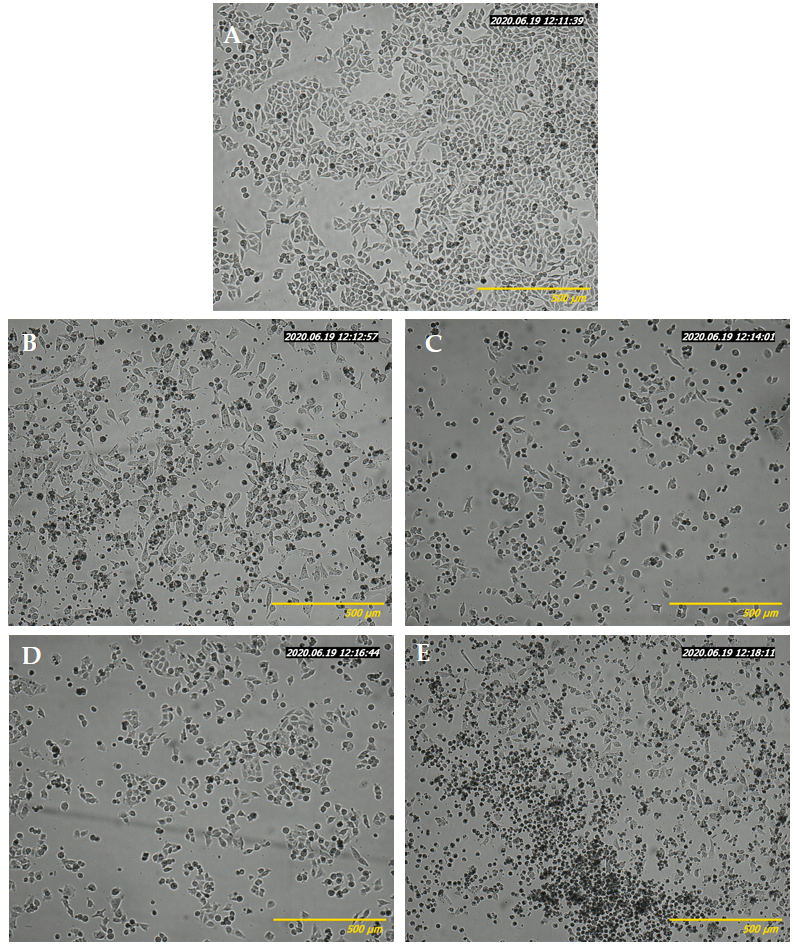

Supplement: Supplementary file 1 [file ijms-22-02462-s001.zip › Supplementary Materials/Figure S3.tif]

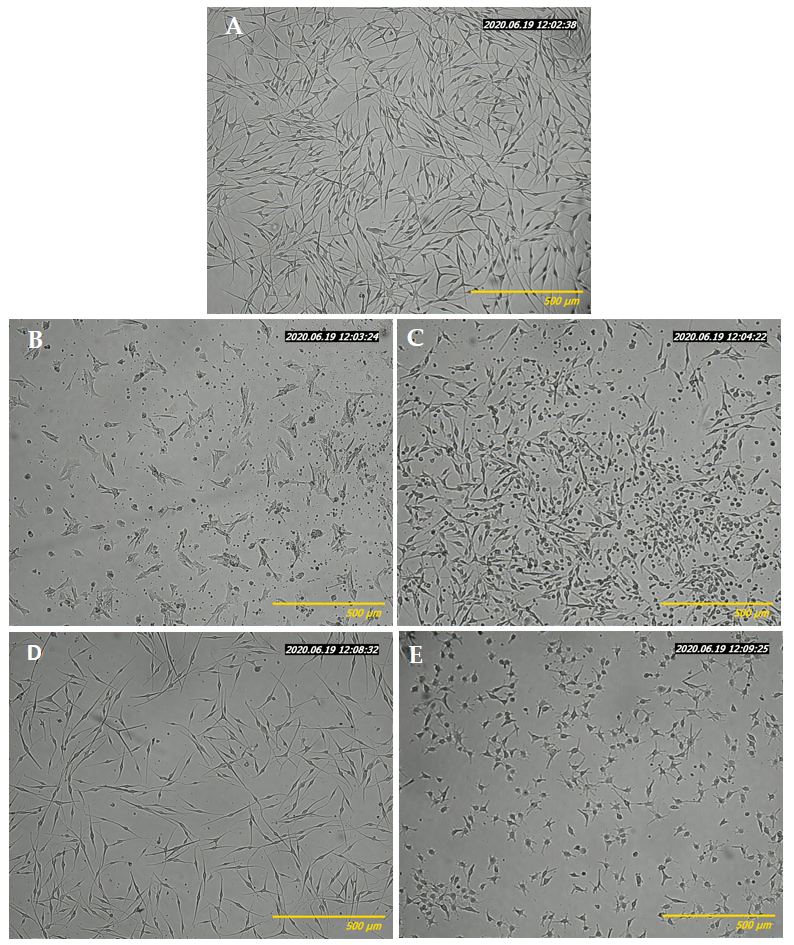

Supplement: Supplementary file 1 [file ijms-22-02462-s001.zip › Supplementary Materials/Figure S4.tif]

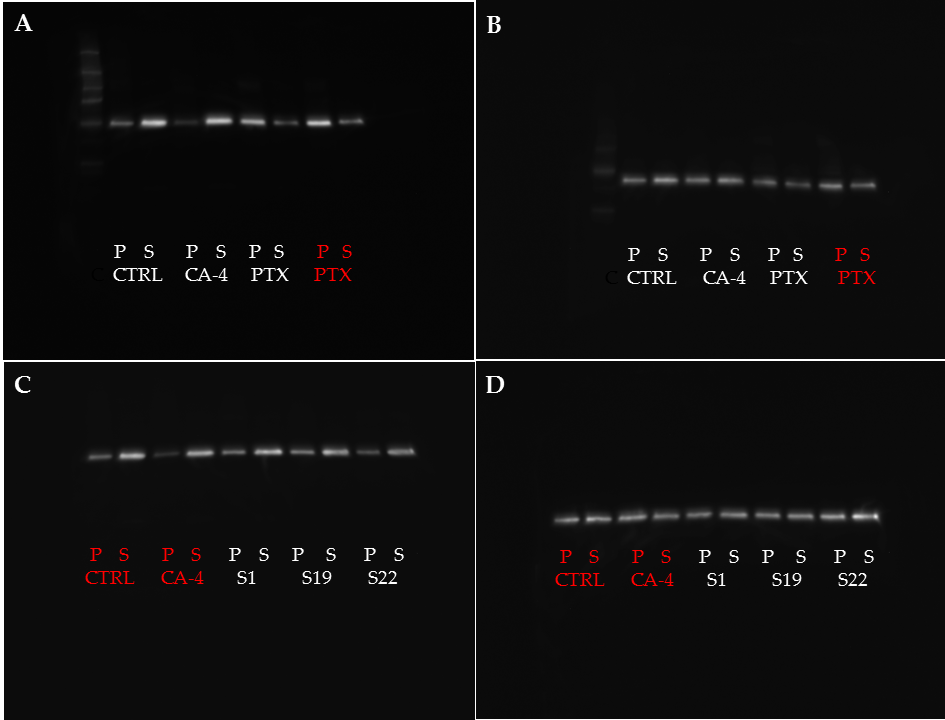

Supplement: Supplementary file 1 [file ijms-22-02462-s001.zip › Supplementary Materials/Figure S6.tif]

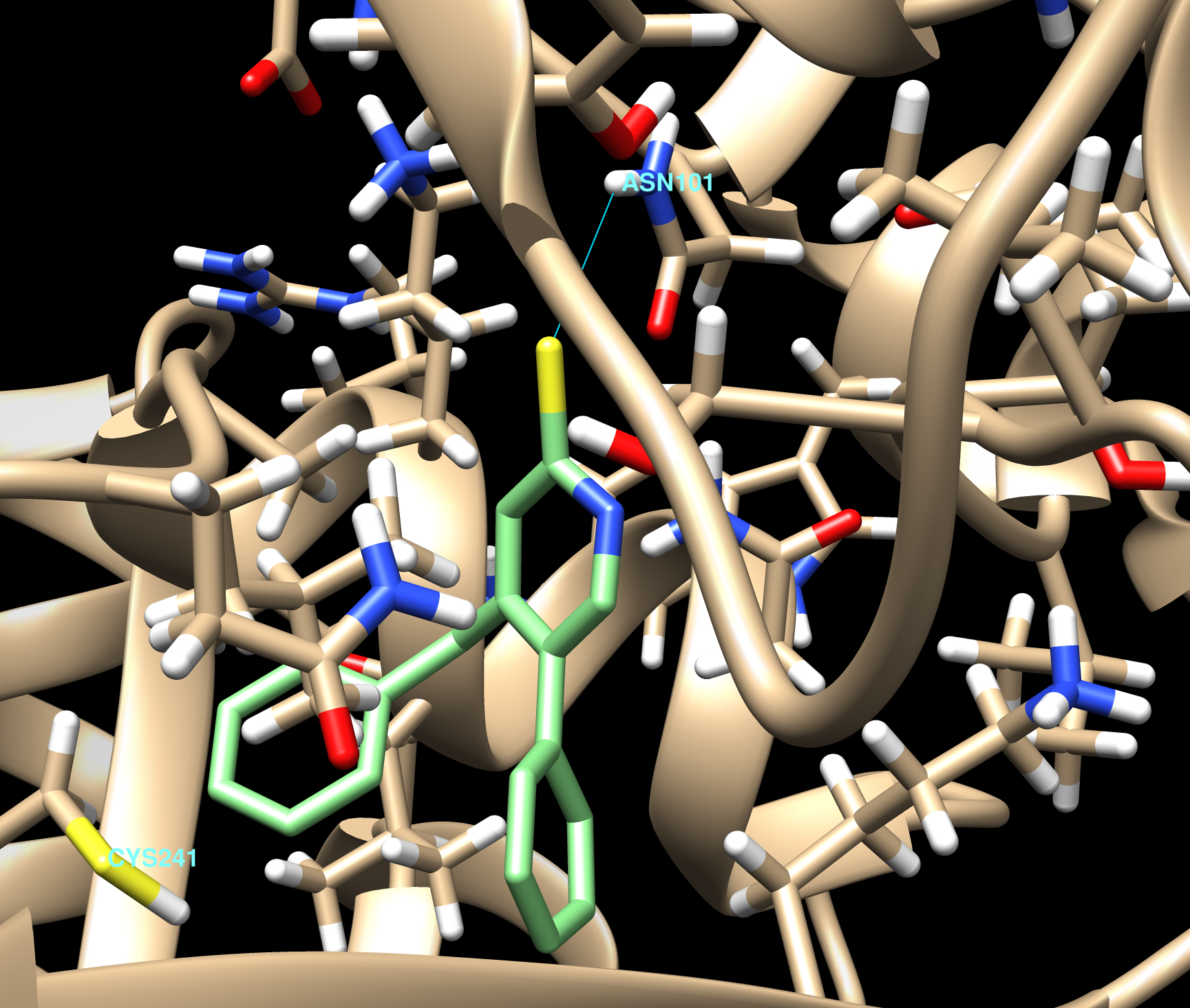

Supplement: Supplementary file 1 [file ijms-22-02462-s001.zip › Supplementary Materials/Figure S7.tif]

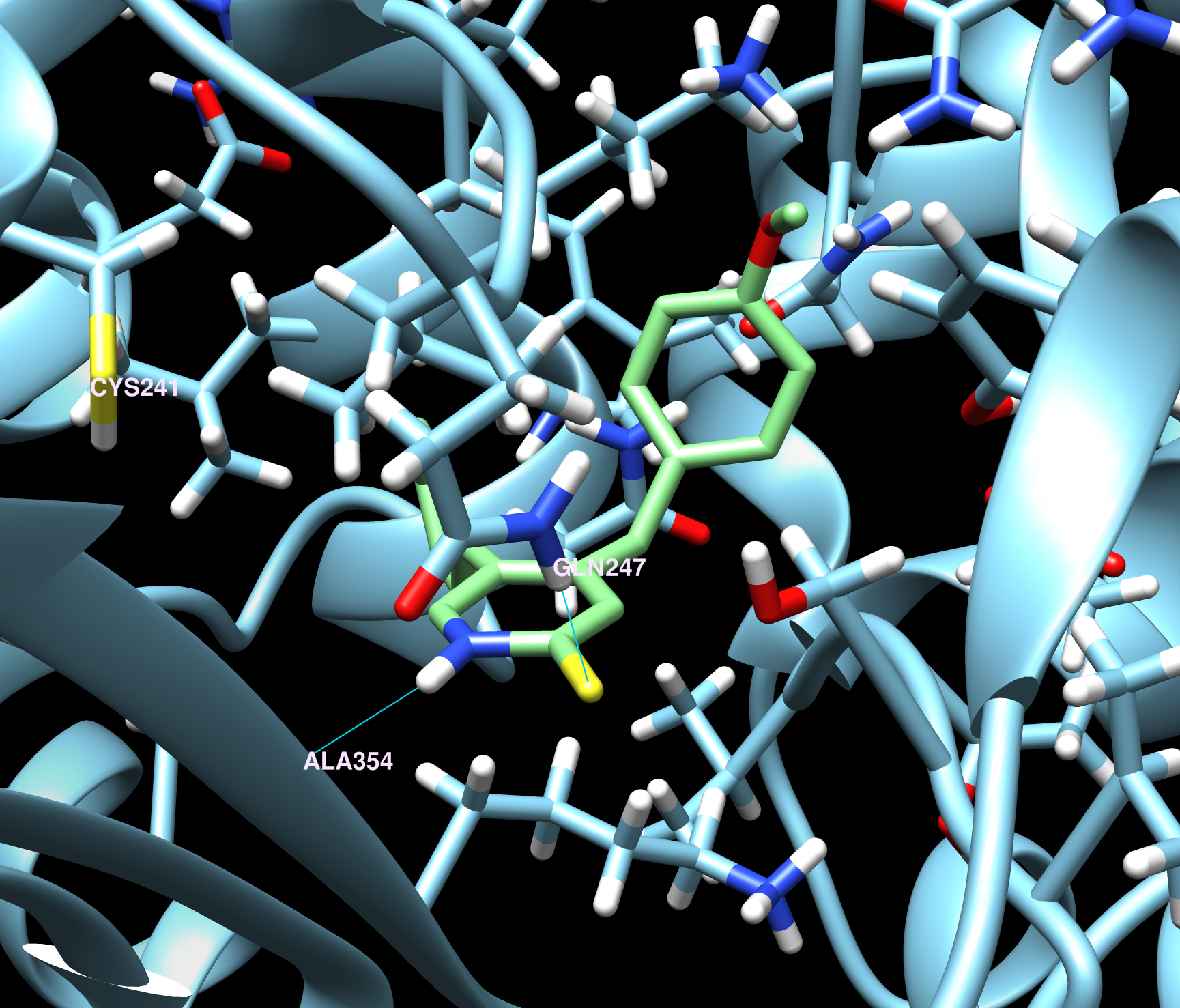

Supplement: Supplementary file 1 [file ijms-22-02462-s001.zip › Supplementary Materials/Figure S8.tif]

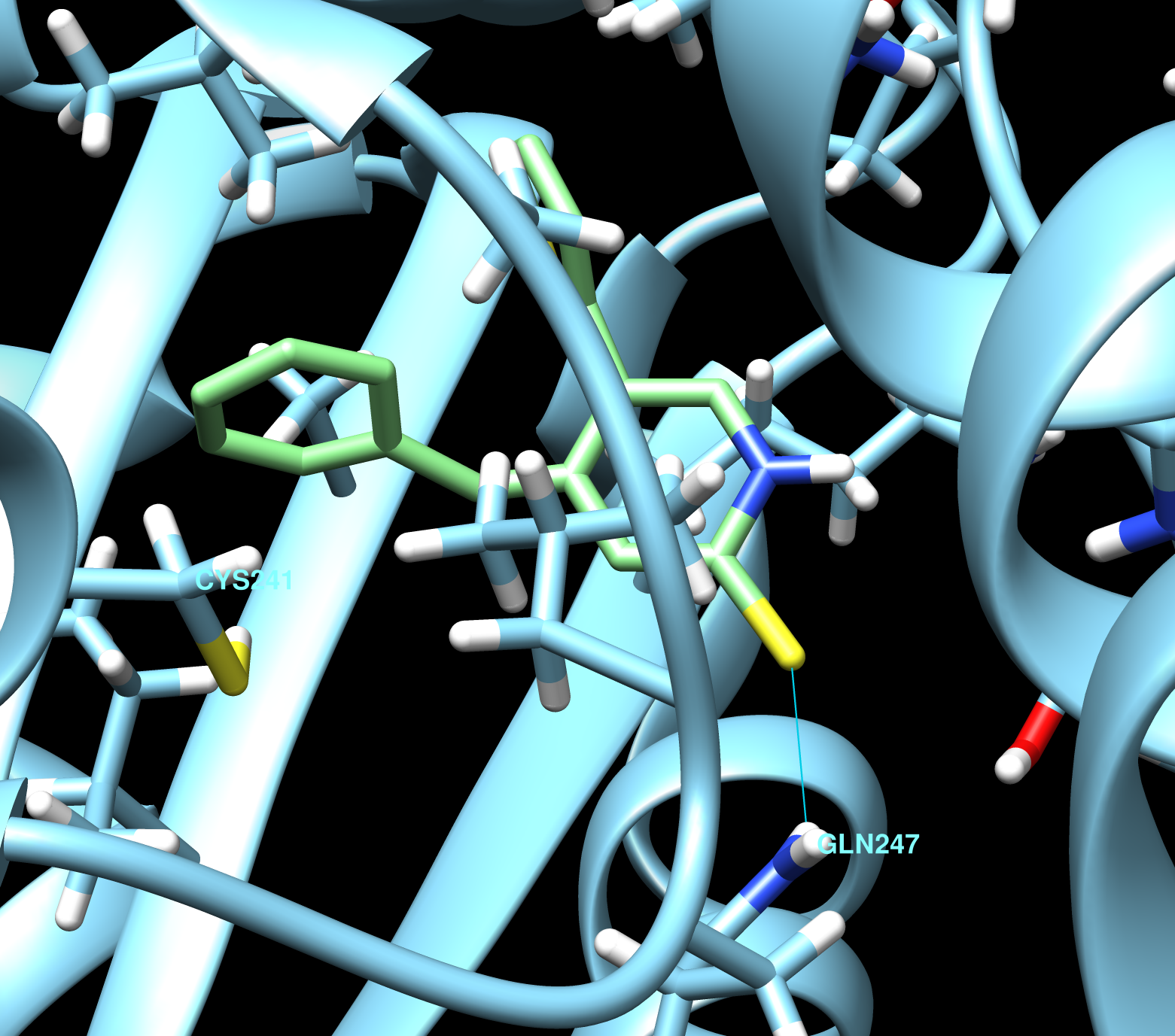

Supplement: Supplementary file 1 [file ijms-22-02462-s001.zip › Supplementary Materials/Figure S9.tif]

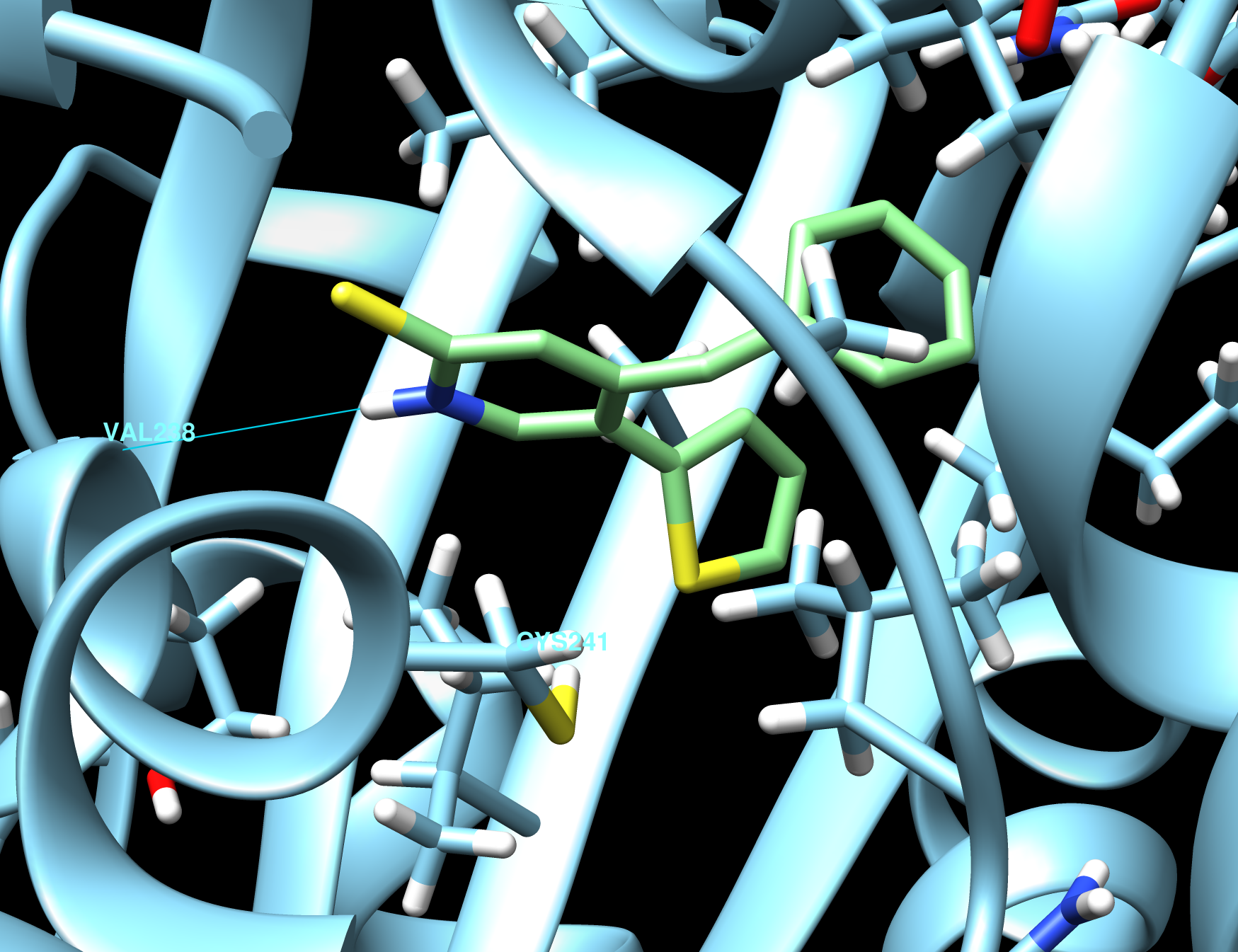

Supplement: Supplementary file 1 [file ijms-22-02462-s001.zip › Supplementary Materials/Figure S10.tif]

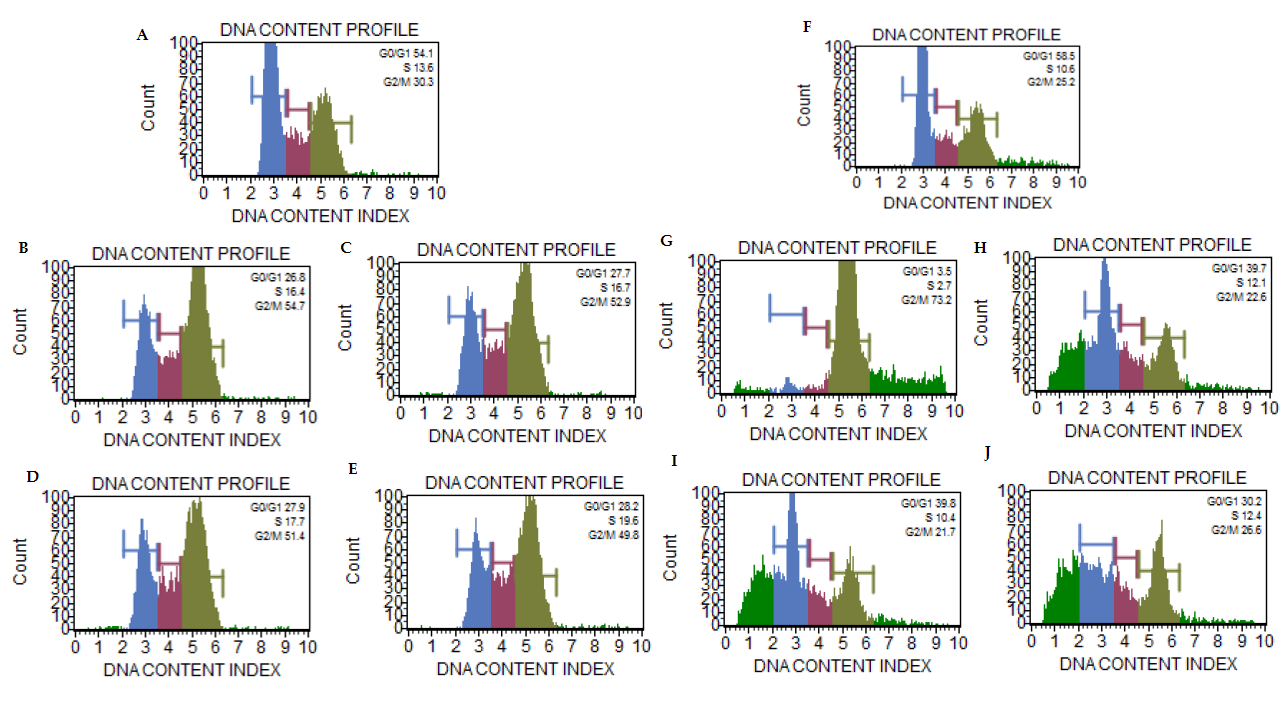

Supplement: Supplementary file 1 [file ijms-22-02462-s001.zip › Supplementary Materials/Figure S5.tif]
